# A new approach towards biomarker selection in estimation of human exposure to chiral chemicals: a case study of mephedrone

**DOI:** 10.1038/s41598-017-12581-3

**Published:** 2017-11-02

**Authors:** Erika Castrignanò, Marie Mardal, Axel Rydevik, Bram Miserez, John Ramsey, Trevor Shine, G. Dan Pantoș, Markus R. Meyer, Barbara Kasprzyk-Hordern

**Affiliations:** 1 Department of Chemistry, University of Bath, Claverton Down, Bath, BA2 7AY United Kingdom; 2 Department of Experimental and Clinical Toxicology, Institute of Experimental and Clinical Pharmacology and Toxicology, Saarland University, Homburg(Saar), 66421 Germany; 3 TICTAC Communications, St George’s University of London, Cranmer Terrace, London, SW170RE United Kingdom

**Keywords:** Environmental monitoring, Diagnostic markers

## Abstract

Wastewater-based epidemiology is an innovative approach to estimate public health status using biomarker analysis in wastewater. A new compound detected in wastewater can be a potential biomarker of an emerging trend in public health. However, it is currently difficult to select new biomarkers mainly due to limited human metabolism data. This manuscript presents a new framework, which enables the identification and selection of new biomarkers of human exposure to drugs with scarce or unknown human metabolism data. Mephedrone was targeted to elucidate the assessment of biomarkers for emerging drugs of abuse using a four-step analytical procedure. This framework consists of: (i) identification of possible metabolic biomarkers present in wastewater using an *in-vivo* study; (ii) verification of chiral signature of the target compound; (iii) confirmation of human metabolic residues in *in-vivo*/*vitro* studies and (iv) verification of stability of biomarkers in wastewater. Mephedrone was selected as a suitable biomarker due to its high stability profile in wastewater. Its enantiomeric profiling was studied for the first time in biological and environmental matrices, showing stereoselective metabolism of mephedrone in humans. Further biomarker candidates were also proposed for future investigation: 4′-carboxy-mephedrone, 4′-carboxy-normephedrone, 1-dihydro-mephedrone, 1-dihydro-normephedrone and 4′-hydroxy-normephedrone.

## Introduction

Wastewater-based epidemiology (WBE) is a new approach that utilises biomarker analysis in wastewater with the aim of understanding, estimating and monitoring populations’ health and lifestyle. WBE is being currently applied to monitor spatial and temporal illicit drug usage at local, national and international scales^1–12^. A list of biomarkers including cocaine, benzoylecgonine, amphetamine, methamphetamine, (±)-3,4-methylenedioxymethamphetamine (MDMA), 11-nor-9-carboxy-delta(9)-tetrahydrocannabinol and other compounds (e.g. heroin, 6-monoacetylmorphine, morphine, mephedrone, ketamine, γ-hydroxybutyric acid- GHB-) has recently been proposed in order to achieve a more comprehensive estimation of drug abuse at community level^13^.

A new compound that was detected for the first time in wastewater via non-targeted high resolution mass spectrometry (HRMS) screening can be considered as a potential biomarker and an indicator of an emerging trend in public health and lifestyle. This is of particular importance in the identification and monitoring of the emergence of new psychoactive substances (NPSs). Unfortunately, it is very difficult to validate new biomarkers mainly due to limited or unavailable human metabolism data. This manuscript presents a new framework that enables the identification and selection of new biomarkers of human exposure to drugs with limited or absent knowledge on human metabolism. Mephedrone was chosen as a target compound in this study to elucidate the assessment of biomarkers for a new emerging drug of abuse using a multi-step analytical procedure.

Mephedrone is a stimulant semisynthetic derivative of cathinone with no licensed medical use. It was first synthesised in 1929 by Saem de Burnaga Sanchez but its abuse was documented for the first time only in 2007^14^. Abuse of mephedrone was reported in several European countries. Recently, several mephedrone abuse associated deaths were reported in the UK^15^. In response to this, several modified cathinones were included in the UK Misuse of Drugs Act (class B) in April 2010. Four fatalities due to mephedrone intake were confirmed in Scotland between February and May 2010^16^.

Mephedrone is a chiral compound. It contains one chiral carbon and it exists in two enantiomeric forms as *R*-(+)-mephedrone and *S*-(−)-mephedrone. Mephedrone can be synthesised via both non-stereoselective and stereoselective methods as shown in Figure S1, but ‘street mephedrone’ is most probably distributed as racemate as reported by the European Monitoring Centre for Drugs and Drug Addiction (EMCDDA)^17^. Routes of administration include oral administration, snorting (nasal inhalation), rectal or intravenous administration. Metabolism of mephedrone in humans and rats was investigated by several research groups^14,18,19^. The metabolism in humans was verified by Pozo *et al*. 2015^20^ using an *in vivo* study in two volunteers. Six phase I and four phase II metabolites were reported in urine (Fig. S2). Normephedrone and 4′-hydroxy-mephedrone, which are two phase I metabolites, showed biological activity serving as substrates at monoamine transporters^21^. Stereoselectivity of mephedrone was hardly investigated. Stereospecific effects of mephedrone enantiomers in rats were reported by Gregg *et al*.^22^. *R*-(+)-mephedrone showed predominant dopaminergic action and more stimulant-like properties than *S*-(−)-mephedrone^23^.

Mephedrone was reported by EMCDDA (EU Early Warning System) to have increased usage in the UK in 2014^24^. Its purity showed a decreasing trend in South Wales since its ban in the UK (from 84.7% in December 2011 to June 2012 to 45.6% in December 2012)^25^. Not surprisingly, it was also detected and quantified in wastewater in Cambridge (UK)^26^ and during a one week monitoring campaign in the UK in 2014^27^. There is very limited information regarding mephedrone in wastewater. While it was found in ten Chinese megacities at levels <2.8 mg/1000 inhabitants day^−1^ ^19^, it was detected in only two Italian cities over a four-year monitoring study, which suggested its low use in Italy^28^. In all studies, the drug target residue (DTR) for WBE estimations was the parent compound mephedrone, due to very limited quantitative information on human metabolism. This constitutes an issue in the WBE approach, as lack of metabolic DTRs does not allow for accurate verification of drug use (e.g. distinction between drug consumption and disposal of unused drug). To solve this problem, this manuscript proposes a novel comprehensive framework that enables biomarker selection in WBE for new drugs of abuse with limited knowledge of human metabolism.

## Results

**Figure 1:**
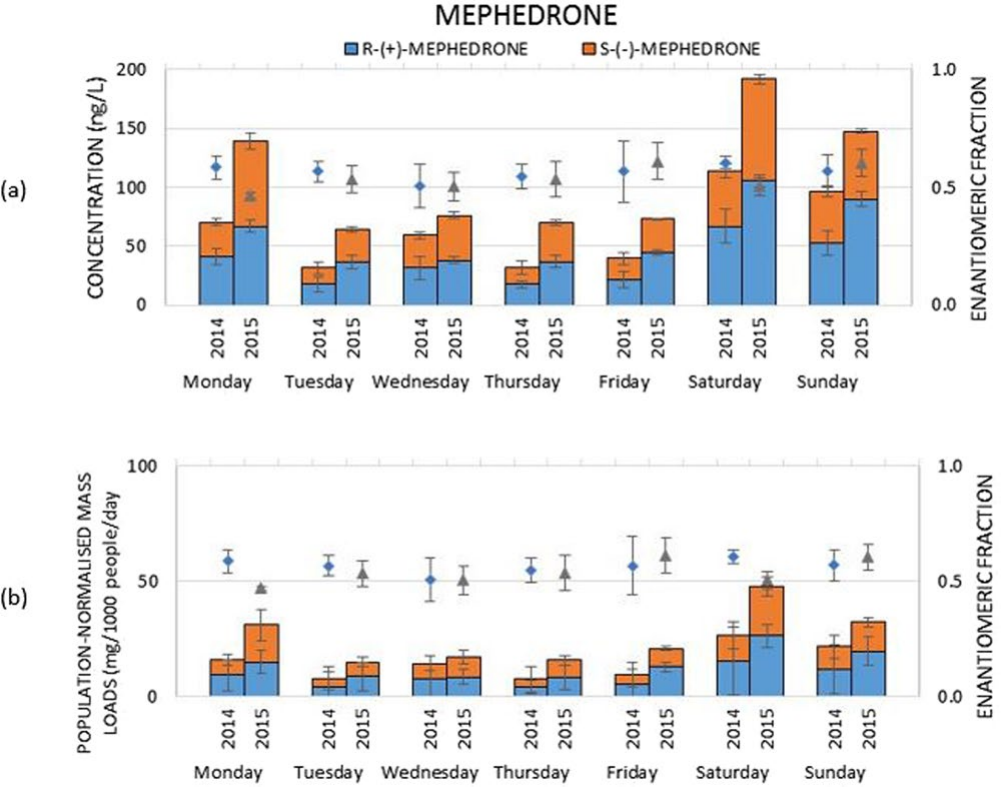
Mephedrone in a week monitoring program. Results are displayed as concentrations (columns) in (**a**), population normalised-loads (columns) in (**b**) and enantiomeric fractions (symbols). The unpaired t-test showed “t Stat > t Critical one-tail” for all wastewater samples excluding Wednesday in 2014 (8.80 > 4.78), p one-tail (0.000024) < 0.001 and for all wastewater samples excluding Monday in 2015 (2.83 > 2.01), p one-tail (0.018) < 0.05. Therefore, EFs from wastewater samples were significantly different (EF > 0.5) from EF = 0.5 during validation. Paired t-test results showed “t Stat < t Critical one-tail” (1.14 < 1.94), p one-tail (0.15) > 0.05, so two datasets of wastewater samples were not significantly different from each other.

**Figure 2:**
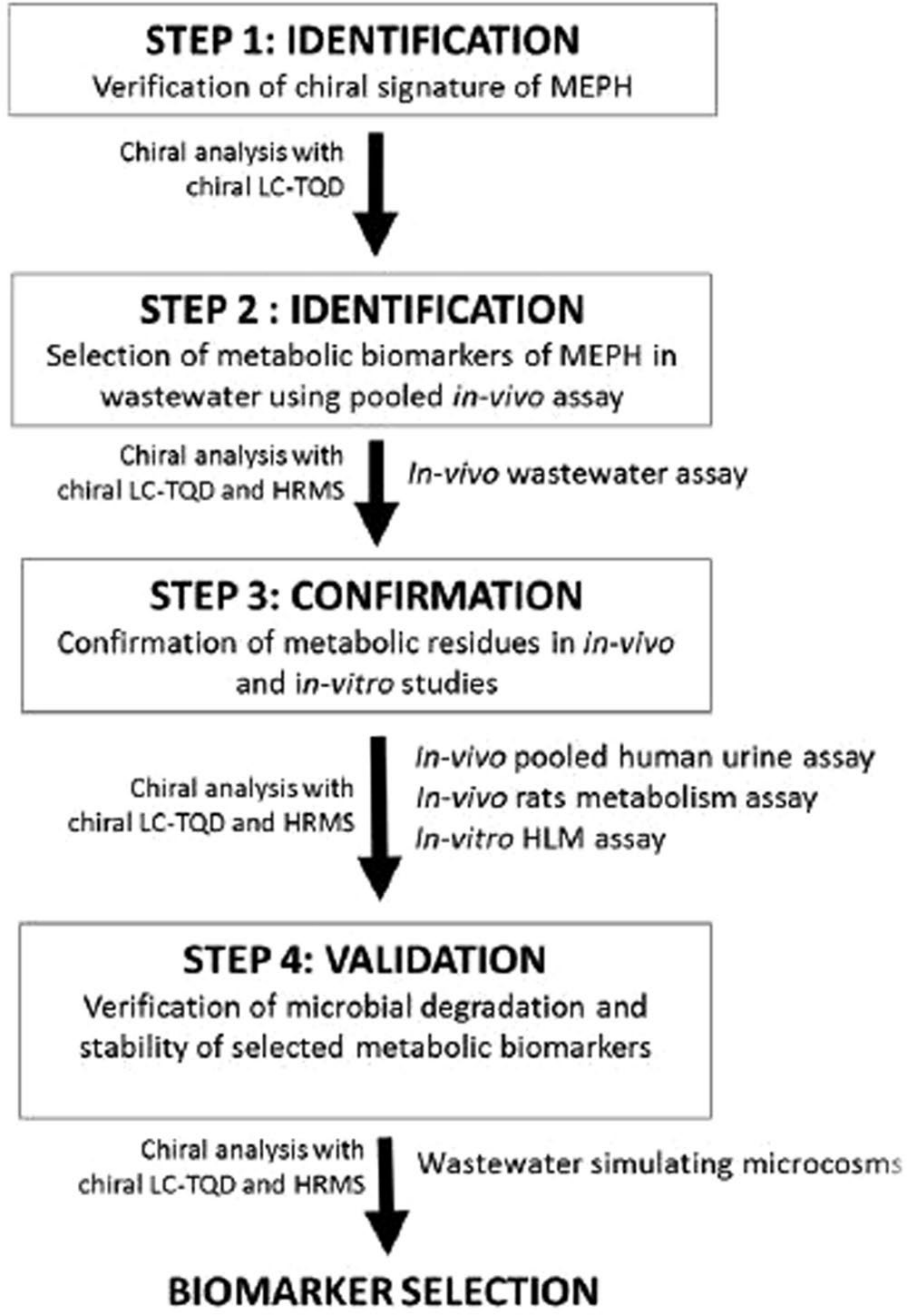
Proposed analytical framework for the identification of suitable biomarkers of new occurring compounds for wastewater-based epidemiology (WBE) approach (MEPH = mephedrone, HLM = human liver microsomes).

Two sampling campaigns undertaken in the UK in 2014 and 2015 confirmed the widespread presence of mephedrone in wastewater. Its concentrations varied from 32 to 114 ng L^−1^ in 2014 and from 65 to 192 ng L^−1^ in 2015 (Fig. 1a and Table S1). Population-normalised mass loads were calculated as described elsewhere^6^. Briefly, daily mephedrone loads (g day^−1^) were obtained by multiplying measured concentrations (ng L^−1^) in daily samples with the corresponding wastewater volumes (L day^−1^). Mephedrone loads were then normalized by the population size of the catchment (mg 1000 people^−1^ day^−1^). Loads ranged from 7.6 to 26.3 mg 1000 people^−1^ day^−1^, with a mean value of 14.7 ± 7.2 mg 1000 people^−1^ day^−1^ in 2014, whilst they ranged from 14.9 to 47.7 mg 1000 people^−1^ day^−1^ with a mean value of 25.6 ± 12.0 mg 1000 people^−1^ day^−1^ in 2015 (Fig. 1b and Table S1). The trend observed for the population-normalised mass loads throughout a week showed the highest loads during the weekend, which suggested its recreational use. As stated by EMCDDA^17^, mephedrone is consumed by users in total doses per session of possibly 500–2000 mg rather than single doses of 5–250 mg, due to short-lived effects. However, even if a mean dose value of 1.25 g was considered, daily doses could not be back calculated using WBE due to missing DTR excretion data. Interestingly, enantioselective analysis revealed that mephedrone present in wastewater was enriched with *R*-(+)-mephedrone, except for the racemate found on Monday and Saturday in 2015 and on Wednesday in 2014 and 2015. This was achieved by the evaluation of the enantiomeric fraction (EF) that is an indicator of the proportion of enantiomers in a mixture. EF is calculated as (+)/[(+) + (−)], therefore it equals 0.5 in the case of 1:1 ratio, or 0 or 1 in the case of enantiomerically pure mephedrone. As reported by EMCDDA^17^, mephedrone is distributed in Europe as racemate. Therefore, the presence of racemate in wastewater can indicate direct disposal. Enrichment of mephedrone with *R*-(+)-enantiomer can indicate stereoselective metabolism in humans and/or stereoselective microbial metabolic processes occurring in wastewater. However, due to lack of data on metabolism of mephedrone in humans and its fate in wastewater, no definite conclusions could be drawn regarding mephedrone abuse in the studied population. Therefore, in this paper, we aimed to propose a robust analytical framework to enable accurate drug abuse estimation via WBE (Fig. 2). The framework consists of four steps:

Step 1: Identification of possible metabolic biomarkers of mephedrone present in wastewater using liquid chromatography coupled to high resolution mass spectrometry, LC-HRMS (pooled *in vivo* study).

Step 2: Verification of chiral signature of mephedrone using chiral liquid chromatography coupled with tandem mass spectrometry (LC-MS/MS).

Step 3: Confirmation of metabolic residues in *in vivo* (human and rat) and *in* vitro (pooled Human Liver Microsomes-pHLM- and phase II conjungation) studies.

Step 4: Microbial degradation in wastewater and verification of stability of possible mephedrone biomarkers in wastewater.

### Step 1: Identification of possible metabolic biomarkers of mephedrone present in wastewater using LC-HRMS

**Figure 3:**
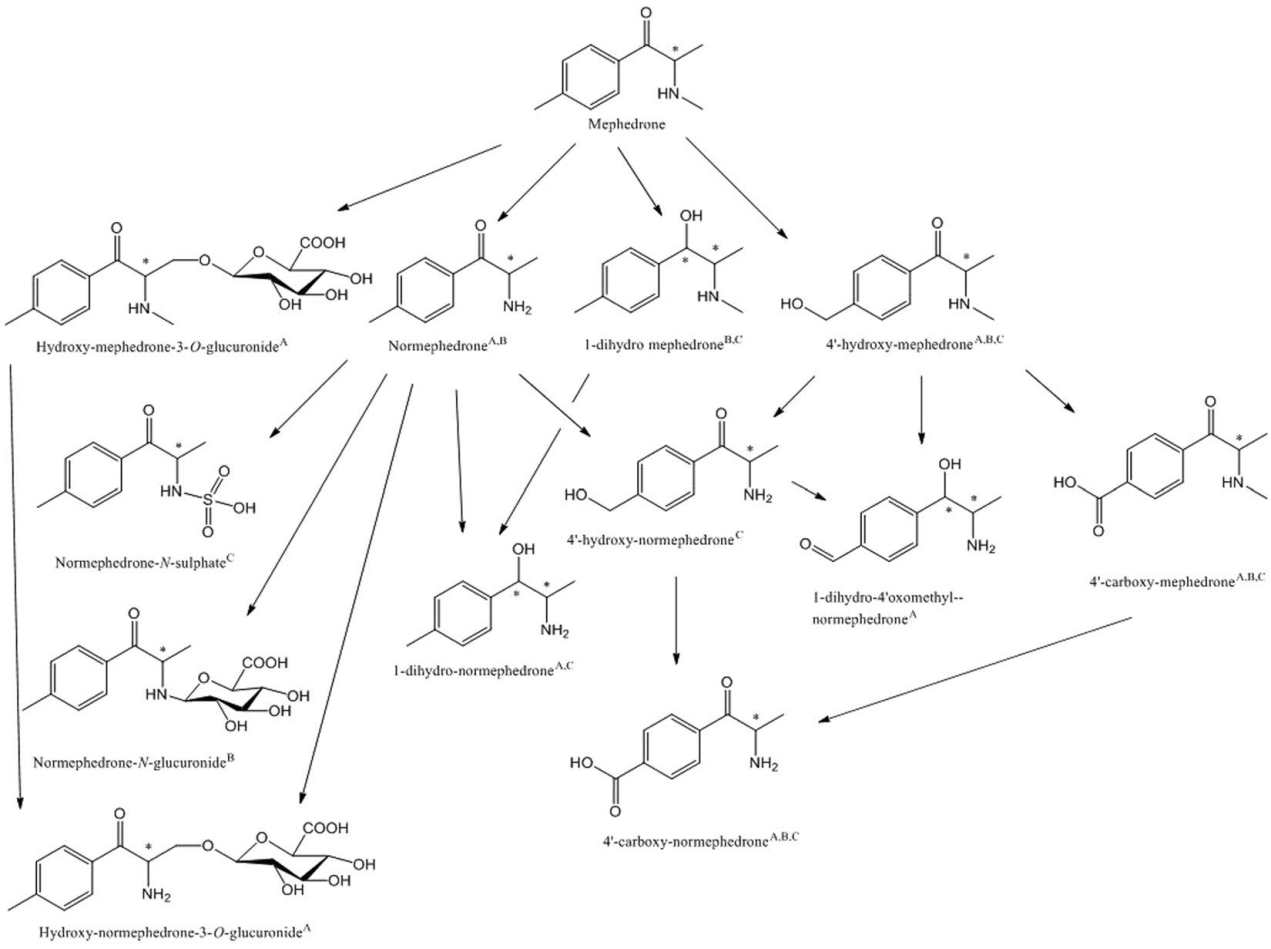
(±)-Mephedrone metabolites detected in the investigated matrices and proposed scheme of metabolism (^A^metabolites found in rat urine, ^B^metabolites found in pHLM study, ^C^metabolites found in wastewater).

A combined approach of targeted and non-targeted LC-HRMS analyses was applied to ensure an effective strategy for metabolic biomarker selection. Target screening analysis of wastewater using liquid chromatography coupled with quadrupole time-of-flight (LC-QTOF) system did not confirm the presence of normephedrone (Table S2). However, non-targeted analysis using Metabolite Identification (MetID) software enabled the prediction and the detection of several metabolites: 1-dihydro-mephedrone, normephedrone-*N*-sulphate, 4′-hydroxy-normephedrone, 4′-carboxy-mephedrone and 4′-carboxy-normephedrone (Tables 1 and S3, Fig. 3). Non-targeted screening allowed the detection of 1-dihydro-normephedrone with good mass accuracy for the precursor and the fragment ion with mass error <5 ppm, and one further fragment ion with an error <10 ppm (Table S4). The precursor ion of the 4′-hydroxy-mephedrone was detected in wastewater at 6.2 min with -3.1 ppm mass error and in pHLM at 2.4 min with <5 ppm mass accuracy for both the parent compound and the fragment ions. This change of retention time could be due to matrix effects or the presence of different isomers. Also no fragment ions in wastewater were found (Table S4). Further studies are therefore required to confirm the presence of 4′-hydroxy-mephedrone in wastewater. 4′-carboxy-mephedrone was identified in wastewater, albeit it was not detected in the pHLM. This was because pHLM does not contain the alcohol dehydrogenases, which are in the cytosol, and, therefore, intermediate hydroxyl groups were likely to be detected in the pHLM experiment, whilst further oxidized products in urine (and in wastewater). Further work is, however, needed to verify whether any of the above biomarkers are suitable DTRs for estimation of mephedrone abuse via WBE.

**Table 1:** Overview of mephedrone and its metabolites through target and non-target screening in all the samples investigated in this study (only target screening was performed in pooled urine).

Analyte	Street mephedrone	Rat urine	Pooled urine	pHLM	Wastewater
Mephedrone	X	X	X	X^b^, X^c^	X
Normephedrone		X		X^b^, X^c^	
1-dihydro-mephedrone				X^c^	X
1-dihydro-normephedrone		X			X
4′-hydroxy-mephedrone		X		X^b^	X
4′-hydroxy-normephedrone^a^					X
1-dihydro-4′-oxomethyl-normephedrone^d^		X			
4′-carboxy-mephedrone		X		X^b^	X
4′-carboxy-normephedrone^e^		X		X^c^	X
Hydroxy-mephedrone-3*O*-glucuronide		X			
Hydroxy-normephedrone-3*O*-glucuronide		X			
Normephedrone-*N*-glucuronide^d^				X^c^	
Normephedrone-*N*-sulphate^d^					X

Legend: ^a^metabolites mentioned in^14^, ^b^those formed in pHLM incubation through set A experiment and ^c^through set B,^d^those not previously published, ^e^those mentioned in^34^.

### Step 2: Verification of chiral signature of mephedrone with chiral liquid chromatography coupled with triple quadrupole system (chiral LC TQD)

Chiral signature of chemicals has already been proven invaluable in WBE in confirming consumption of MDMA^29^, atenolol^30^ and direct disposal of fluoxetine^31^. Chiral signature could also prove invaluable in the verification of potency of ‘street drugs’ as well as their synthetic routes. However, in order to apply such an approach, the following two aspects need to be verified: (i) enantiomeric signature of distributed drug and (ii) possible changes in its enantiomeric signature during human metabolism.

Step 2(a): ‘Street’ mephedrone

**Figure 4:**
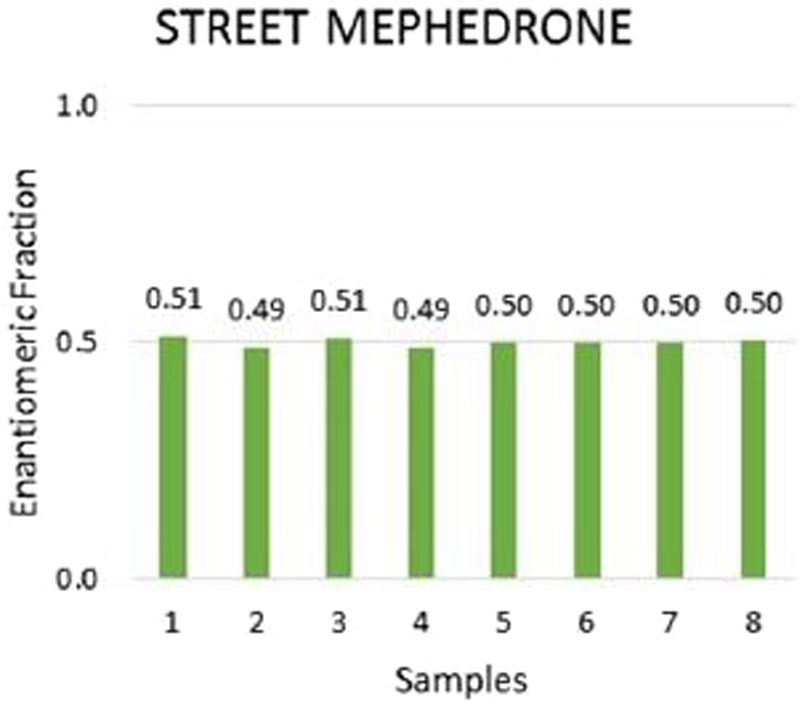
Enantiomeric fraction of mephedrone in illegal samples of mephedrone. The unpaired t-test showed “t Stat < t Critical one-tail” (1.78 < 2.16), p two-tail (0.097) < 0.05. Therefore, EFs from street mephedrone samples were not significantly different from EF = 0.5 during validation.

Chiral LC TQD analysis of eight illegal mephedrone samples resulted in EF averaging at 0.50 ± 0.01 (Fig. 4), which indicates a non-stereoselective method of synthesis. Indeed, this confirms the conclusions of Gibbons and Zloh^32^ and the EMCDDA report on mephedrone^17^.

Step 2(b): Metabolism of mephedrone in humans

As can be seen in Fig. 1, mephedrone quantified in wastewater (with chiral LC-TQD) was enriched with *R*-(+)-mephedrone. Knowing that mephedrone is distributed as racemate, it suggests that the presence of mephedrone in wastewater must have been subject to metabolic processes either in humans or other species such as microbes. Unfortunately, as there is very limited knowledge of stereoisomerism of mephedrone, no conclusions could be drawn without further studies. We therefore applied a multi-step approach in order to verify (a) the stereoselective metabolism of mephedrone in humans and (b) the stereoselective microbial metabolic processes occurring in wastewater (see “step 3”). As it is difficult to undertake *in vivo* metabolism studies of new abused drugs in humans, we tested if *in vitro* experiments utilising pHLM represented a valid and alternative method for metabolism investigation, especially for new designer drugs^33^. We therefore compared our pHLM results with biological samples from animal samples (rat urine) and pooled human urine samples collected at festivals.

### Step 3: Confirmation of metabolic residues in *in vivo* and *in vitro* studies

Step 3(a): *In vitro* metabolism of mephedrone using pHLM.

**Figure 5:**
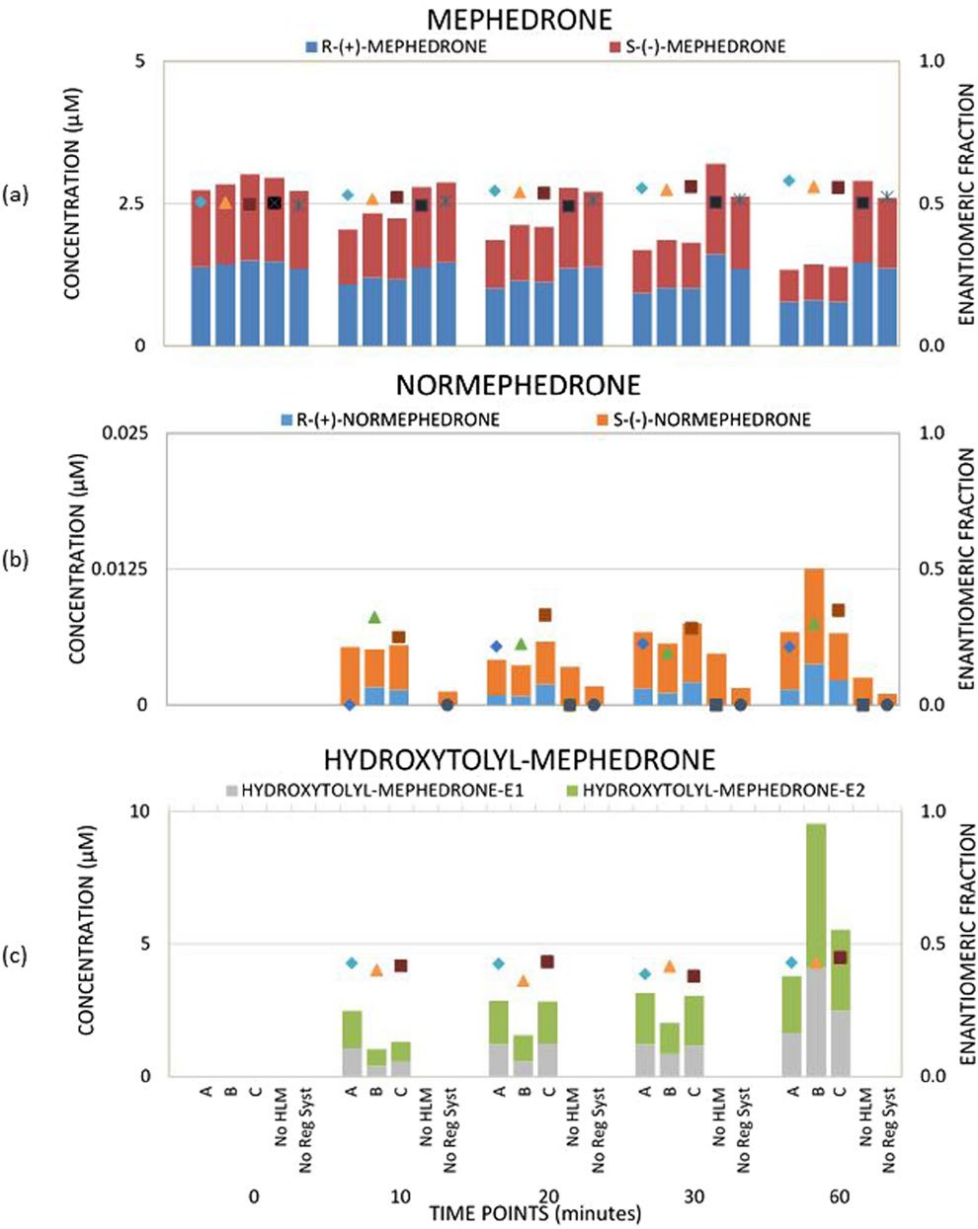
Stereoselective metabolism of mephedrone (**a**) and formation of its metabolites, normephedrone (**b**) and hydroxytolyl-mephedrone (4′-hydroxy-mephedrone) (**c**), in *in vitro* pHLM study. “A, B, C” are incubation reactors and “No HLM, No Reg Syst” are control reactors. All biological pHLM reactors are described in details in Table S7b. Symbols show EF values.

*In vitro* experiments were performed by incubating mephedrone in pHLM to verify the formation of phase I and phase II metabolites (experiment A and B in S2, respectively). pHLM were previously used by Pedersen *et al*.^14^, where they indicated CYP2D6 to be the main enzyme responsible for the microsomal metabolism of mephedrone (this was obtained by using cDNA-expressed cytochrome P450 (CYP) enzymes). In the current study, the results obtained using chiral LC-TQD revealed a stereoselective metabolism of mephedrone leading to an enrichment of mephedrone with *R*-( + )-enantiomer (Fig. 5) and formation of two metabolites: normephedrone and 4′-hydroxy-mephedrone enriched with *S*-(−)-enantiomers.

LC-QTOF non-targeted screening enabled the prediction and the detection of a number of metabolites using MetID software (Tables 1 and S3, Fig. 3). Indeed, normephedrone and a phase I metabolite, 4′-carboxy-normephedrone, were predicted with good mass error (<5 ppm), whilst others were predicted with a mass error <10 ppm for the precursor ion (1-dihydromephedrone, 4′-hydroxy-mephedrone, 4′-carboxy-mephedrone and a new phase II metabolite normephedrone-*N*-glucuronide). Regarding the latter metabolite, glucuronic acid was found to be conjugated to normephedrone *N*- group, in contrast to the 3*O*-glucuronide observed in Pozo *et al*.^20^. Target screening analysis by LC-QTOF further confirmed the presence of normephedrone (Table S2).

Step 3(b): *In vivo* metabolism of mephedrone in rat urine

The following mephedrone metabolites were detected in rat urine sample using LC coupled with a Q Exactive system (LC Q E) (Table S5): normephedrone, 4′-hydroxy-mephedrone, 1-dihydro-normephedrone, 4′-carboxy-mephedrone, 4′-carboxy-normephedrone, hydroxy-mephedrone-3*O*-glucuronide, hydroxy-normephedrone-3*O*-glucuronide, that were previously described by Pedersen *et al*. 2013, Khreit *et al*. 2013 and Linhart *et al*. 2016^14,18,34^ and normephedrone-glucuronide.

**Figure 6:**
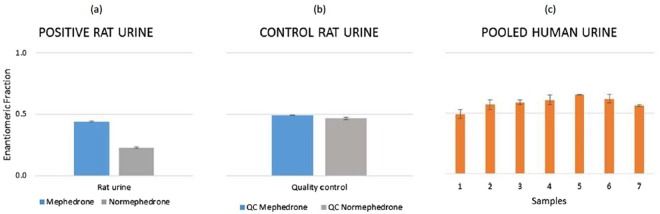
Enantiomeric fraction of mephedrone in’ positive’ (**a**) and in ‘control’ rat urine samples (**b**). Enantiomeric fraction of mephedrone in pooled human urine samples (**c**), where the unpaired t-test showed “t Stat > t Critical one-tail” (4.88 > 1.89 for α = 0.05; 4.88 > 4.78 for α = 0.001), p one-tail (0.00089) < 0.001. Therefore, EFs from pooled human urine samples were significantly different from EF = 0.5.

The analysis of mephedrone excreted in the rat urine with Chiral LC coupled with Orbitrap system Velos Pro (LC VP) revealed that mephedrone undergoes stereoselective metabolism. Indeed, mephedrone and formed metabolites normephedrone (Fig. S3), 4′-hydroxy-mephedrone (Fig. S4) and 1-dihydro-4′oxomethyl-normephedrone (Fig. S5) were not racemic in excreted urine. Interestingly, diastereoisomers of 1-dihydro-normephedrone were also found (Fig. S4) and a predominance of one diastereoisomer with respect to the other was observed in this study. Linhart *et al*. (2016) observed a ratio of 3:1 for *erythro*- and *threo*- 1-dihydro-normephedrone^34^. This proportion among isomers might also be confirmed in this study by assuming the same eluting profile, although their analytical standards were not available. Indeed, EFs were 0.49 ± 0.0 and 0.47 ± 0.0 for mephedrone and normephedrone in spiked control rat urine (Fig. 6b), respectively, whilst a decrease in EF (0.44 ± 0.0 and 0.22 ± 0.0 for mephedrone and normephedrone respectively) was observed in positive rat urine (Fig. 6a). This finding shows stereoselective metabolism of both compounds favouring *S*-(−)-enantiomer. This is in contrast to pHLM studies (see “step 3a”) and human pooled urine (see “step 3c”). Hence, this study indicates that mephedrone metabolism in humans and rats might follow different stereoselective patterns. A possible hypothesis for this finding might be the hydroxylation reaction of *R-*(+)-normephedrone which leads to enrichment of mephedrone with *S*-(−)-normephedrone enantiomer. Moreover, in Figure S5, four peaks showing identical fragmentation patterns indicated the presence of a molecule with two chiral centres. These were identified as 1-dihydro-4′oxomethyl-normephedrone enantiomers as a result of a partial oxidation of the 4′-hydroxy-mephedrone to aldehyde combined with a reduction of the phenone.

Step 3(c): Mephedrone in pooled urine

Chiral LC TQD analysis of pooled human urine samples confirmed predominance of *R*-(+)-mephedrone (Fig. 6c), hence its stereoselective metabolism. This was in agreement with wastewater samples and the pHLM study, indicating that mephedrone in wastewater resulted primarily from its consumption on most days and not direct disposal.

To sum up, non-targeted screening with LC-QTOF and LC Q E confirmed stereoselective metabolism of mephedrone in humans and the presence of several metabolites in wastewater, rat urine and in pHLM samples. These were: 4′-carboxy-mephedrone, 4′-carboxy-normephedrone, 1-dihydro-mephedrone (not in pooled human urine according to chiral analysis), 1-dihydro-normephedrone (not in pHLM) and 4′-hydroxy-normephedrone. Interestingly, normephedrone was not detected in wastewater nor in pooled urine samples.

### Step 4: Microbial degradation in wastewater and verification of stability of possible biomarkers of mephedrone in wastewater

Incubation of mephedrone in wastewater over 24 hours did not lead to formation of transformation products, even when wastewater spiked with mephedrone was incubated in a week long experiment (see Method section).

Stability of DTRs in wastewater is critical if low uncertainty measurements of community-wide drug abuse using WBE are to be undertaken. Good biomarkers need to be stable for at least 24 hours (to ensure stability during 24-h composite sampling time). As recommended by the consensus best practise protocol for sampling and storage in Castiglioni *et al*.^13^, low temperature settings reduce the degradation of biomarkers and help the preservation of the analytes in the samples. Indeed, our results after incubation of wastewater at differing experimental conditions (see Method section) confirmed that low temperature (4 °C) reduces degradation of mephedrone with only approximately 10% change at 4 °C when compared to up to 50% change at 17 °C (Table 2, Fig. S6). Furthermore, no formation of new metabolites was observed, which confirms stability of mephedrone in wastewater over a time of 24 h at 4 °C. In contrast, normephedrone was found to degrade at selected temperature settings.

**Table 2:** Stability of targeted compounds in influent wastewater samples stored over a 48 h (^a^expressed as difference in percentage from time-point 0 ± SD).

Analyte	Stability^a^ [%]			
	Raw (unfiltered) wastewater, pH 7.4, stored at 17 °C		Raw (unfiltered) wastewater, pH 7.4, stored at 4 °C	
	12 h	24 h	12 h	24 h
*R*-(+)-Mephedrone	−23.4 ± 2.7	−29.3 ± 4.7	−9.5 ± 2.7	−10.9 ± 3.5
*S*-(−)-Mephedrone	−7.1 ± 4.8	−10.2 ± 7.0	−4.0 ± 4.0	−11.9 ± 17.1
*R*-(+)-Normephedrone	−19.0 ± 13.6	n.a.^b^	n.a.^b^	−19.9 ± 25.7
*S*-(−)-Normephedrone	−6.9 ± 0.0	1.5 ± 5.1	n.a.^b^	−11.1 ± 1.0

^b^Due to lack of reproducibility and stability of normephedrone in wastewater, values could not be established (for further details see Fig. S6).

## Discussion

This paper proposes a new investigative framework for the selection and validation of metabolic biomarkers of abused drugs (such as NPSs) where limited information on their human metabolism exists, with the ultimate goal of their application in WBE to estimate community-wide drug use. Mephedrone was chosen as a target chemical due to its widespread abuse in the UK, frequent occurrence in wastewater and there being little understanding of its quantitative metabolic profile.

The developed framework consisted of four steps and resulted in the following conclusions:

*Step 1:* *Identification of possible metabolic biomarkers of mephedrone present in wastewater using LC-HRMS* (*pooled in vivo study*).

Several metabolites of mephedrone and potential metabolic biomarkers were identified in wastewater. These were: 1-dihydro-mephedrone, normephedrone-*N*-sulphate, 4′-hydroxy-normephedrone, 4′-carboxy-mephedrone, 4′-carboxy-normephedrone and 1-dihydro-normephedrone.

*Step 2: Verification* of *chiral signature of mephedrone using chiral LC-MS/MS*.

‘Street’ mephedrone was found to be distributed in the UK as racemate.

*Step 3:* *Confirmation of human metabolic residues in in vivo* (*pooled urine*) *and in vitro* (*pHLM*) *studies*.

Stereoselective metabolism of mephedrone favouring *R-*(+)*-*enantiomer was observed in pHLM experiments. This was further confirmed by pooled urine analysis. Interestingly, *in vivo* rat metabolism studies led to contrasting results where *S-*(−)*-*mephedrone was favoured. This questions rat studies as a stand-alone approach towards biomarker selection for WBE.

*In vitro* pHLM experiments lead to identification of several metabolites and potential biomarkers of mephedrone abuse. Remarkably, normephedrone formed via stereoselective metabolism of mephedrone in *in vitro* pHLM studies was not identified in pooled urine samples. This might be due to dilution of pooled urine samples with urine from non-abusers and degradation probably occurring during the time the pooled urine was in the metal tank, prior to collection, at outside temperature (25 < T(°C) < 29).

*Step 4:* *Microbial degradation in wastewater and verification of stability of possible biomarkers of mephedrone in wastewater*.

Wastewater simulating microcosms revealed high stability of mephedrone with up to a week long incubation time at 4 °C.

In the light of the above evidence, the following conclusions were drawn:

- Mephedrone is a suitable candidate as a biomarker, because of its high stability in wastewater and stereoselective metabolism in humans.
- Chiral analysis is fundamental in the enantiomeric profiling of mephedrone, especially in distinguishing between human consumption and direct disposal of unused drug. This could be further proved by in-sewer transport studies.
- Despite stereoselective formation of normephedrone in *in vitro* pHLM studies, this metabolite was found to be unsuitable as a biomarker of mephedrone consumption as (i) it was detected in neither pooled human urine nor wastewater and (ii) it has low stability in wastewater.
- Possible biomarker candidates (apart from mephedrone) for further investigations are: 4′-carboxy-mephedrone, 4′-carboxy-normephedrone, 1-dihydro-mephedrone, 1-dihydro-normephedrone and 4′-hydroxy-normephedrone.

## Methods

### Materials

Table S6 shows target analytes, their properties and supplier information. Mephedrone-D_3_ was used as an internal standard (IS). All standards were of the highest purity available (≥98%). Stock and working solutions of standards were stored at −20 °C. Methanol, acetonitrile and ammonium acetate were purchased from Sigma Aldrich, UK. Ultrapure water was obtained from a PURELAB UHQ-PS Unit (Elga, UK). The deactivation of the glassware was carried out according to^27^. Commercial pHLM were purchased from Sigma Aldrich, UK. Glucuronic acid (CAS 6556-12-3, Sigma Aldrich, UK) and active sulphate adenosine 3′-phosphate 5′-phosphosulfate lithium salt hydrate (PAPS-CAS 109434-21-1, Sigma Aldrich, UK) were used as substrates for the investigation of the mephedrone phase II metabolism.

### Sample collection and preparation

#### Street mephedrone samples

Eight street mephedrone powder samples were collected from amnesty bins at a festival in the UK in 2014. Methanolic solutions were prepared and stored in a freezer at −20 °C. Diluted solutions in 1 mM ammonium acetate/methanol 85:15 v/v (MP_CBH_) were spiked with IS solution at 1 μg mL^−1^ and injected in the chiral LC TQD system.

#### Rat urine samples

According to the usual study design^18^, the investigations on mephedrone metabolism in rats were performed using rat urine samples from male Wistar rats (Charles River, Germany) for toxicological diagnostic reasons according to the corresponding German law (http://www.gesetze-im-internet.de/tierschg/). Rat faeces and urine samples were separated during the 24 hours of collection time and stored at −20 °C in a freezer. Rat urine samples collected before drug administration were used as control samples. Collected urine samples were diluted 100-fold and directly injected into a LC-HRMS system: LC Q E (Thermo Fisher Scientific, MA, USA). Acetylation of rat urine was carried out to verify the presence of hydroxyl groups in mephedrone metabolites as described in S1. To undertake chiral LC TQD analysis, samples were reconstituted in 100 μL of MP_CBH_.

All methods were carried out in accordance with the corresponding German law (Tierschutzgesetz, animal protection act, https://www.gesetze-im-internet.de/tierschg/BJNR012770972.html). All experiments were licensed by the Dezernat V (Sicherheit und Ordnung) under K 110/180-07 (Anzeige von Versuchvorhaben nach § 8a Abs. 1 und 2 TierSchG).

#### Pooled urine samples

Seven pooled urine samples (from five different urinals sampled on three different days) were collected in August 2014 from a UK festival event. Pooled urine samples were collected anonymously from a pool contributed to by a large number of anonymous donors from a very large population of over 70,000 people. The project was approved by University of Bath Department of Chemistry Ethics Committee and TICTAC Communications. 3 mL of each sample were spiked with 50 μL of IS at 1 μg mL^−1^ and underwent solid-phase extraction (SPE) using Oasis HLB cartridges (60 mg, Waters, UK) as described in^27^. Liquid-liquid extraction was then performed using ethyl acetate and sodium phosphate at pH 8-9. Samples were centrifuged for 5 minutes at 5000 rpm. The supernatant was evaporated to dryness under nitrogen flow at 40 °C and reconstituted in 250 μL of MP_CBH_. After filtration through 0.2 µm PTFE filters (Whatman, Puradisc, 13 mm), 20 μL were injected on the chiral LC TQD system.

#### Wastewater samples

*Monitoring campaigns*. 24 h time-proportional (10 mL every 15 minutes) composite wastewater samples were collected in PTFE bottles from a local wastewater treatment plant. They were then transported to the laboratory in cool boxes packed with ice blocks and filtered through GF/F 0.7 µm glass fibre filters (Whatman, UK). Sample preparation was performed as described in^27^. 20 µL of samples were directly injected into the chiral LC TQD system.

*Stability of mephedrone in wastewater*. Stability of mephedrone and normephedrone in wastewater was investigated in dark biotic conditions at 4 °C and 17 °C for a duration of 48 hours. 500 mL of wastewater were spiked in duplicate with 1 μg L^−1^ of mephedrone or normephedrone. Unspiked wastewater reactors were also included as controls. 50 mL of wastewater samples were collected at 0, 12, 24 and 48 hours, and spiked with IS. pH and temperature were constantly monitored. Samples were prepared according to^27^. Eluates were dried and reconstituted in 0.5 mL of MP_CBH_ for chiral LC TQD analysis and in 0.5 mL of methanol for LC Q E analysis.

*Incubation of mephedrone in wastewater and formation of microbial transformation products*. In accordance with a previously published procedure, using QuEChERs sample preparation method^35^, mephedrone was incubated in four reactors: biotic (containing wastewater spiked with the analyte), abiotic (containing the wastewater spiked with the analyte and sodium azide to quench any bacterial growth), clean (containing demineralised water spiked with the analyte and sodium azide) and control (only wastewater) (Table S7a). Sampling was performed at 0, 4 and 7 days. Samples were then injected on the LC Q E system.

In order to detect biotransformation products of the incubated wastewater, SPE was performed using Biotage HCX cartridges as follows: conditioning with 1 mL of methanol and 1 mL of deionised water; loading of 3 mL of filtered and spiked with IS wastewater; washing with 1 mL of deionised water, 1 mL of 0.01 M HCl and 1 mL deionised water. 2 mL of methanol were used for eluting the neutral fraction, whilst 1 mL methanol/NH_4_OH 33% 98:2 v/v was used for the basic fraction. Analysis of data dependent MS/MS fragmentation (ddMS2) was performed. The software systems EAWAG-BBD Pathway Prediction System (http://eawag-bbd.ethz.ch/predict/) and XCMS Online by the Scripps Research Institute (https://xcmsonline.scripps.edu/) were used.

#### pHLM experiments

Two experiments were performed for the *in vitro* metabolism studies of mephedrone in accordance with previously published procedures^36,37^. In the first one (A), mephedrone was incubated at a concentration considered more “realistic” for abusers focusing on phase I metabolites only, whilst in the second (B), a higher concentration was used for enhancing the metabolites identification (Table 7b). Both experimental settings are described in the Supplementary Material S2.

### Sample analysis with LC-MS/MS

#### Quantification of mephedrone and its metabolites using targeted quantitative analysis and chiral LC TQD analysis

Separation of all the analytes was undertaken with the validated methodology using chiral LC TQD system according to^27^. Source setting is described in the Supplementary Material (S3). MassLynx (Waters, UK) was used to control the Waters ACQUITY system and the Xevo TQD. Data processing was carried out on TargetLynx 4.1 software (Waters, UK). MRM transitions, cone voltages and collision energies are summarised in Table S8. In Table S9 validation parameters, such as instrumental and method limits of detection and quantification (IDL, IQL, MDL, MQL), linearity (Table S9a), SPE recovery (Table S9b), method precision (Table S9c), instrumental precision (Table S9d), resolution of enantiomers and EF (Table S9e) are shown. The analytes showed linearity from 0.25 µg L^-1^ up to 500 µg L^-1^ for single enantiomer highlighting the high level of performance of cellobiohydrolase (CBH) column for these compounds. IDL and MDL were 0.25 µg L^-1^ for both analytes and nearly 1 ng L^-1^, whilst IQL and MQL were in both cases lower for mephedrone than for normephedrone. The identification criteria for each analyte were in accordance to European guidelines^38^.

#### Identification of metabolites using targeted and non-targeted analysis with LC-HRMS

The analyses were performed using three different HRMS systems: LC-VP, LC-Q E and LC-QTOF respectively.

*Orbitrap system Velos Pro* (*LC-VP*). The separation of the analytes was undertaken with an UltiMate 3000 HPLC system (Thermo Fisher Scientific, MA, USA) and a CHIRALPAK^®^ CBH HPLC Column with a Chiral-CBH guard column. The UltiMate 3000 HPLC autosampler was kept at 4 °C, while the column compartment at 25 °C. The chromatographic conditions were described in^27^. The Orbitrap Velos Pro (Thermo Fisher Scientific, MA, USA) was equipped with a heated ESI source (H-ESI) operating in positive mode. Data processing was carried out on Xcalibur 2.1 software (Thermo Fisher Scientific, MA, USA).

*Q Exactive* (*LC-Q E*). The LC–HRMS system was composed of a Thermo Fisher Scientific (MA, USA) Accela LC system consisting of a degasser, a quaternary pump and an HTC PAL autosampler (CTC Analytics AG, Switzerland), an Accucore™ Phenyl-Hexyl HPLC Column 2.6 μm particle size, L × I.D. 10 cm × 2.1 mm (Thermo Fisher Scientific, MA, USA) coupled to a Thermo Fisher Scientific Q Exactive system, equipped with a HESI-II source operated in positive and negative ion modes. LC and MS source setting are described in S3. Data were acquired in full-scan mode and a subsequent data dependent acquisition mode over a mass range of 130–1000 *m/z* with a resolving power of 35000 FWHM, microscans of 1, automatic gain control (AGC) target at 1e^6^, maximum injection time (IT) of 120 ms. Data processing was carried out by using Xcalibur 2.1 software (Thermo Fisher Scientific, MA, USA).

*QTOF system* (*LC-QTOF*). MaXis High-Definition (HD) Q-TOF system (Bruker, Daltonik GmbH, Germany) was equipped with an ESI source operating in positive and negative mode. It was coupled to an UltiMate 3000 UPLC system (Thermo Fisher Scientific, MA, USA) and an ACQUITY UPLC BEH C18 Column 5 cm 2.1 mm × 1.7 µm particle size (Waters, UK). The UltiMate 3000 UPLC autosampler was kept at 4 °C, while the column compartment at 40 °C. LC and MS source setting are described in S3. Bruker Compass DataAnalysis v.4.3 was the controlling software for the analyses. HRMS data processing was carried out on ACD/Labs MetID software. It allowed the research of metabolites generated by the regioselectivity algorithms of ACD/Percepta, matching with those present in the experimental dataset and identifying them through IntelliTarget Algorithm.

The absolute configuration determination of mephedrone using circular dichroism (CD) and computational study is described and discussed in S4, Table S10, Figures S7 and S8.

All above methods were performed in accordance with the relevant guidelines and regulations.

## Electronic supplementary material

Supplementary material
