# Publisher Correction: A new approach towards biomarker selection in estimation of human exposure to chiral chemicals: a case study of mephedrone

**DOI:** 10.1038/s41598-017-18350-6

**Published:** 2017-12-22

**Authors:** Erika Castrignanò, Marie Mardal, Axel Rydevik, Bram Miserez, John Ramsey, Trevor Shine, G. Dan Pantoș, Markus R. Meyer, Barbara Kasprzyk-Hordern

**Affiliations:** 1 Department of Chemistry, University of Bath, Claverton Down, Bath, BA2 7AY United Kingdom; 2 Department of Experimental and Clinical Toxicology, Institute of Experimental and Clinical Pharmacology and Toxicology, Saarland University, Homburg(Saar), 66421 Germany; 3 TICTAC Communications, St George’s University of London, Cranmer Terrace, London, SW170RE United Kingdom

Correction to: *Scientific Reports* 10.1038/s41598-017-12581-3, published online 02 November 2017

The original PDF version of this Article contained a typographical error in the publication date ‘2^nd^ November 2017’ which was incorrectly given as ‘11^th^ October 2017’. This has now been corrected in the PDF version of the Article.

