# Publisher Correction: A new approach towards biomarker selection in estimation of human exposure to chiral chemicals: a case study of mephedrone

**DOI:** 10.1038/s41598-018-22367-w

**Published:** 2018-03-06

**Authors:** Erika Castrignanò, Marie Mardal, Axel Rydevik, Bram Miserez, John Ramsey, Trevor Shine, G. Dan Pantoș, Markus R. Meyer, Barbara Kasprzyk-Hordern

**Affiliations:** 1 Department of Chemistry, University of Bath, Claverton Down, Bath, BA2 7AY United Kingdom; 2 Department of Experimental and Clinical Toxicology, Institute of Experimental and Clinical Pharmacology and Toxicology, Saarland University, Homburg(Saar), 66421 Germany; 3 TICTAC Communications, St George’s University of London, Cranmer Terrace, London, SW170RE United Kingdom

Correction to: *Scientific Reports* 10.1038/s41598-017-12581-3, published online 02 November 2017

An error occurred after publication resulting in the inadvertent omission of the Article and Volume numbers from the PDF version of this Article. This error has now been corrected in the PDF version of the Article; the HTML version was correct from the time of publication.

