# Supplementary material for: A new approach towards biomarker selection in estimation of human exposure to chiral chemicals: a case study of mephedrone
Source: Sci Rep. 2017 Nov 2;7:13009. doi: 10.1038/s41598-017-12581-3 (PMC5693930; doi:10.1038/s41598-017-12581-3)
Supplement: Supplementary file 1 — Supplementary material [file 41598_2017_12581_MOESM1_ESM.pdf]

A new approach towards biomarker selection in estimation of human exposure to chiral chemicals: a case study of mephedrone

Erika Castrignanò, Marie Mardal, Axel Rydevik, Bram Miserez, John Ramsey, Trevor Shine, G. Dan Pantoş, Markus R. Meyer, B. Kasprzyk-Hordern

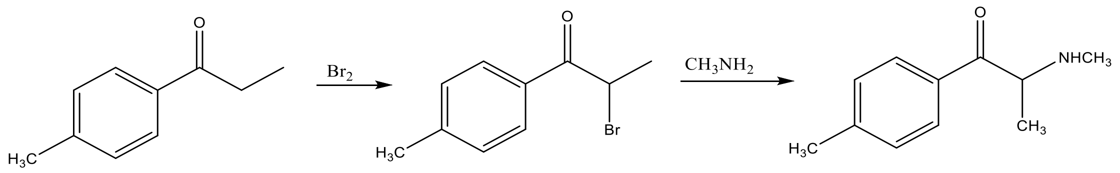

(a)

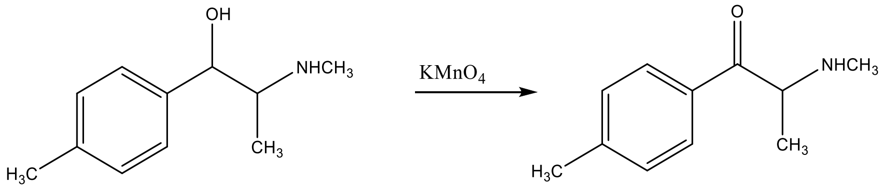

(b)

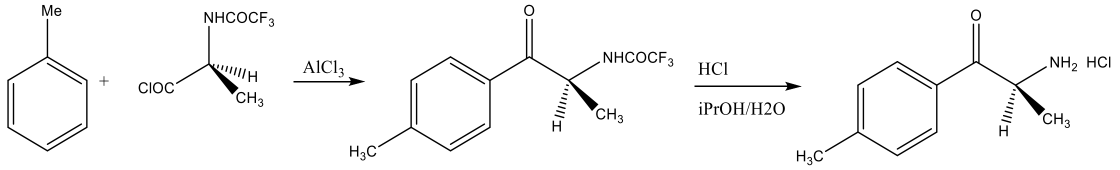

(c)

Figure S1 Synthesis of (±)-mephedrone (a, b) (modified from Schifano et al. (2011) (Schifano *et al.*, 2011) and of *S*-(-)-mephedrone (c) (modified from Osorio-Olivares et al. (2003) (Osorio-Olivares *et al.*, 2003).

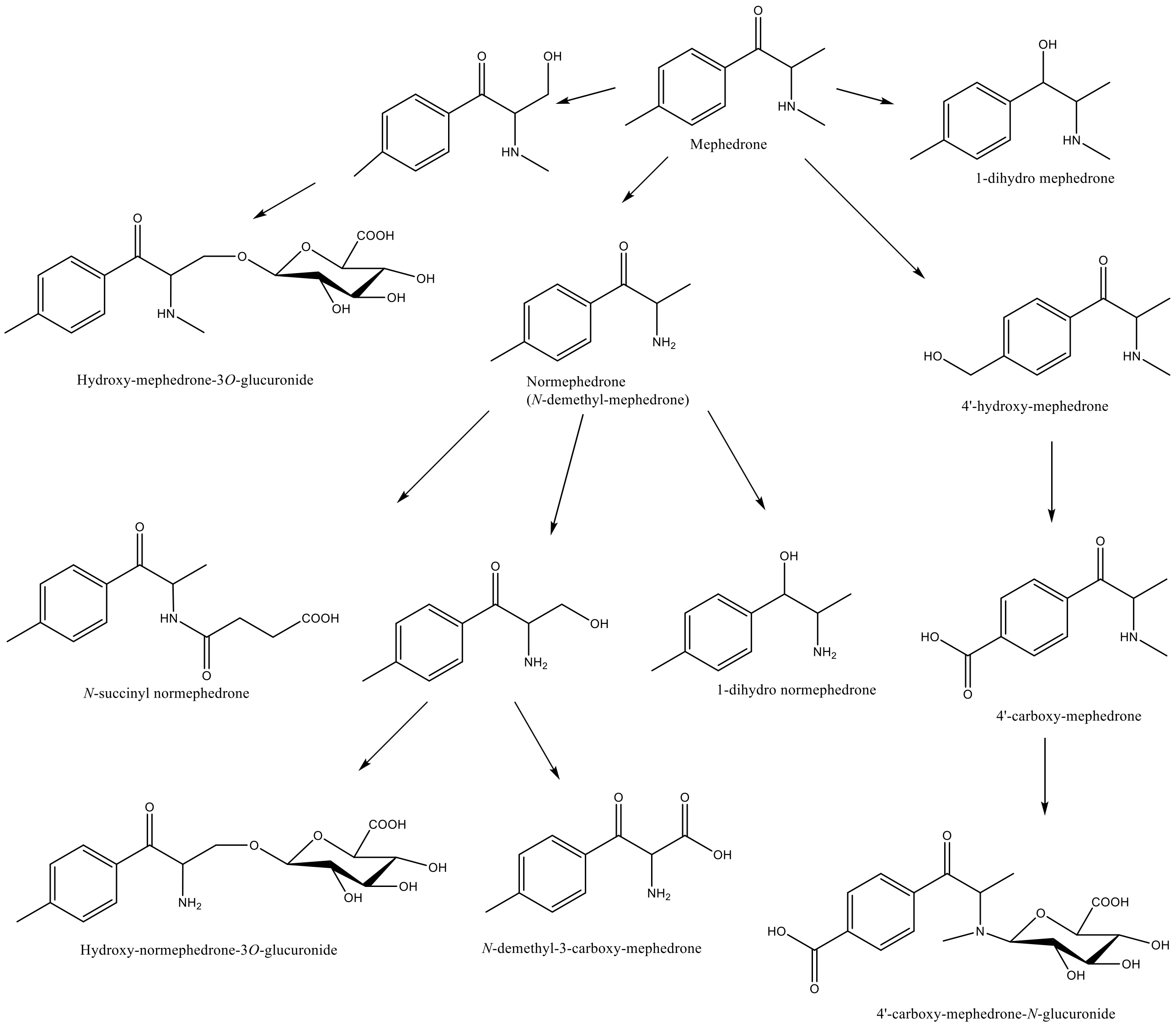

Figure S2 Proposed mephedrone metabolism in humans (modified from Pozo et al. (2014)).

Table S1 Mephedrone concentrations and population-normalised mass loads in wastewater samples during one week monitoring campaign in 2014 (from 11th to17th March) and in 2015(from 10th to16th March) in the UK ([a] Population Equivalent).

**2014**								
**Day**	**Flow [m³ day⁻¹]**	**PE[a]**	***R*-(+)-Mephedrone**		***S*-(-)-Mephedrone**		**(±)-Mephedrone**	
			Concentration [ng L⁻¹]	Population-normalised mass loads [mg 1000 people⁻¹day⁻¹]	Concentration [ng L⁻¹]	Population-normalised mass loads [mg 1000 people⁻¹day⁻¹]	Concentration [ng L⁻¹]	Population-normalised mass loads [mg 1000 people⁻¹day⁻¹]
Monday	199012	886650	42 ± 7	9.3	29 ± 2	6.5	70.5	15.8
Tuesday	216049	886650	18 ± 6	4.3	14 ± 5	3.4	31.8	7.7
Wednesday	214229	886650	32 ± 10	7.7	28 ± 3	6.6	59.3	14.3
Thursday	208782	886650	18 ± 3	4.2	14 ± 6	3.4	32.3	7.6
Friday	208644	886650	22 ± 7	5.1	18 ± 5	4.3	40.0	9.4
Saturday	204287	886650	67 ± 15	15.4	47 ± 6	10.8	114.0	26.3
Sunday	198221	886650	53 ± 11	11.8	44 ± 5	9.8	96.5	21.6
Average				8.3		6.4		14.7
SD				4.2		3.0		7.2
CV				0.5		0.5		0.5
**2015**								
**Day**	**Flow [m³ day⁻¹]**	**PE[a]**	***R*-(+)-Mephedrone**		***S*-(-)-Mephedrone**		**(±)-Mephedrone**	
			Concentration [ng L⁻¹]	Population-normalised mass loads [mg 1000 people⁻¹day⁻¹]	Concentration [ng L⁻¹]	Population-normalised mass loads [mg 1000 people⁻¹day⁻¹]	Concentration [ng L⁻¹]	Population-normalised mass loads [mg 1000 people⁻¹day⁻¹]
Monday	197493	886650	67± 5	14.9	72 ±7	16.1	140	31.1
Tuesday	204491	886650	37 ± 6	8.5	27 ± 2	6.3	65	14.9
Wednesday	198950	886650	38 ± 3	8.5	38 ± 3	8.5	76	17.1
Thursday	197523	886650	37 ± 5	8.2	33 ± 2	7.4	70	15.6
Friday	252682	886650	45 ± 2	12.7	28 ± 1	8.1	73	20.8
Saturday	220687	886650	106 ± 5	26.3	86 ± 4	21.4	192	47.7
Sunday	193194	886650	90 ± 6	19.6	58 ± 2	12.5	148	32.1
Average				14.1		11.5		25.6
SD				6.8		5.6		12.0
CV				0.5		0.5		0.5

Table S2 Target screening analysis in wastewater and in pHLM by using LC-QTOF (*PI* means precursor ion, *DI* daughter ion).

**STANDARD MEPHEDRONE-D3**							
	**In mobile phase**			**In wastewater**		**In pHLM**	
**Chromatogram**	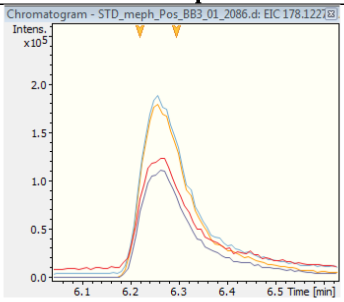			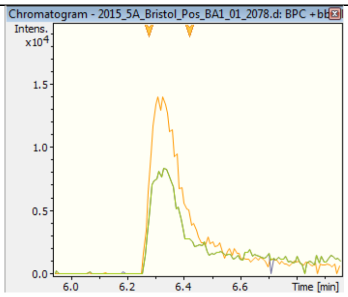		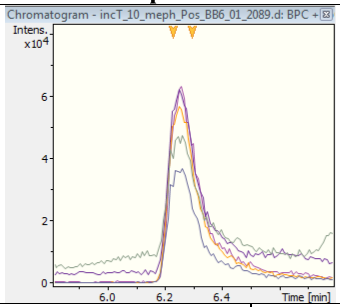	
	**Theor. mass [M+H]$^+$**	**Experimental mass [M+H]$^+$**	**Mass Error (ppm)**	**Experimental mass [M+H]$^+$**	**Mass Error (ppm)**	**Experimental mass [M+H]$^+$**	**Mass Error (ppm)**
**PI**	181.1415	181.1417	1.10	181.1408	-3.86	181.1416	0.55
**DI**	163.1309	163.1310	0.61	163.1303	-3.68	163.1310	0.61
**MEPHEDRONE**							
	**In mobile phase**			**In wastewater**		**In pHLM**	
**Chromatogram**	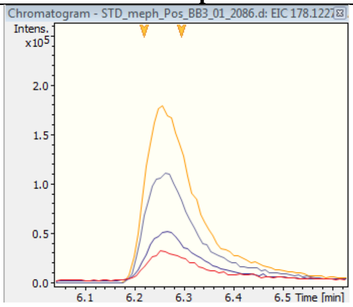			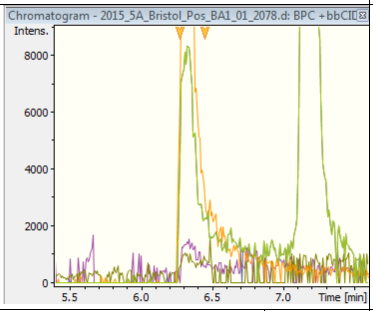		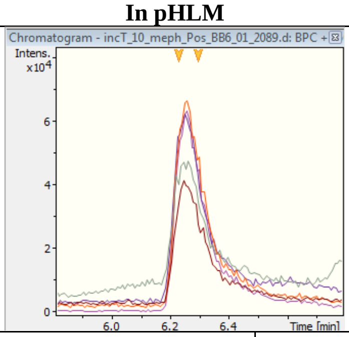	
	**Theor. mass [M+H]$^+$**	**Experimental mass [M+H]$^+$**	**Mass Error (ppm)**	**Experimental mass [M+H]$^+$**	**Mass Error (ppm)**	**Experimental mass [M+H]$^+$**	**Mass Error (ppm)**
**PI**	178.1226	178.1227	0.56	178.1219	-3.93	178.1228	1.12
**DI**	160.1121	160.1120	-0.62	160.1115	-3.75	160.1121	0
**NORMEPHEDRONE**							
	**In mobile phase**			**In wastewater**		**In pHLM**	
**Chromatogram**	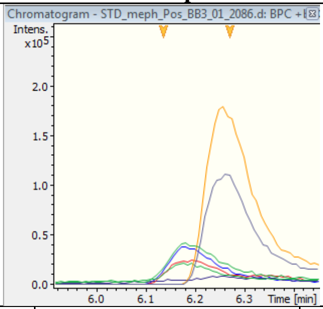			-		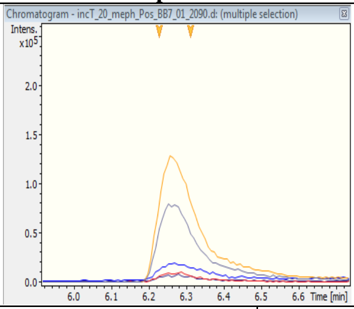	
	**Theor. mass [M+H]$^+$**	**Experimental mass [M+H]$^+$**	**Mass Error (ppm)**	**Experimental mass [M+H]$^+$**	**Mass Error (ppm)**	**Experimental mass [M+H]$^+$**	**Mass Error (ppm)**
**PI**	164.1070	164.1071	0.61	-	n.d.	164.1070	0
**DI**	147.0804	147.0804	0	-	n.d.	147.0807	2.04
**DI**	119.0855	119.0854	-0.84	-	n.d.	119.0857	1.68

Table S3 Non-targeted analysis by LC Q-TOF: mephedrone metabolites predicted in wastewater and in pHLM by using MetID software (*Theor* means theorethical and *Exp.* experimental).

**Metabolite**	**Formula**	**Ionization mode**	**Rt**	**Precursor ion**		
				**Theor. mass ($[M+H]^+$ or $[M-H]^{-}$)**	**Exp. mass ($[M+H]^+$or $[M-H]^{-}$)**	**Mass Error (ppm)**
Wastewater						
Dihydro-mephedrone	C11H17NO	ESI +	5.50	180.1383	180.1384	0.55
Nor-hydroxy-tolyl-mephedrone	C10H13NO2	ESI -	6.70	178.0874	178.0871	-1.68
4'-carboxy mephedrone	C11H13NO3	ESI -	5.85	206.0823	206.0822	0.48
4'-carboxy normephedrone	C10H11NO3	ESI -	6.92	192.067	192.066	0.52
Normephedrone-*N*-sulphate	C10H13NO4S	ESI +	7.15	244.0640	244.0638	0.82
pHLM						
Normephedrone	C10H13NO	ESI +	6.18	164.1070	164.1064	3.65
4'-carboxy-normephedrone	C10H11NO3	ESI -	6.90	194.0812	194.0820	4.12

Table S4 Untarget screening analysis in wastewater and in pHLM by using LC-QTOF.

1-DIHYDRO-NORMEPHEDRONE					
	**Structure**	**In wastewater**		**In pHLM**	
**Chromatogram**	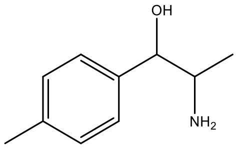	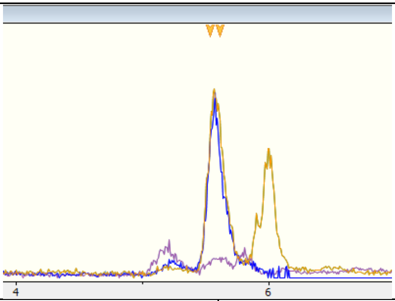		-	
	**Theor. mass [M+H]$^+$**	**Experimental mass [M+H]$^+$**	**Mass Error (ppm)**	**Experimental mass [M+H]$^+$**	**Mass Error (ppm)**
**PI**	1166.1226	166.1223	-1.80	-	-
**DI**	1148.1121	148.1119	-1.35	-	-
**DI**	115.0542	115.0528	-8.70	-	-

4'-HYDROXY-MEPHEDRONE					
	**Structure**	**In wastewater**		**In pHLM**	
**Chromatogram**	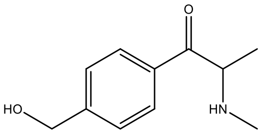	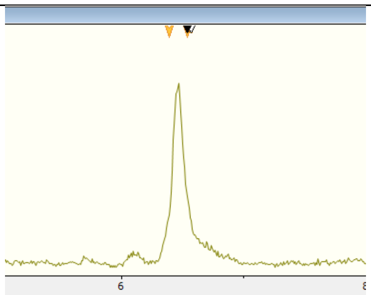		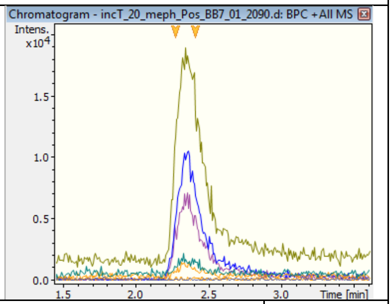	
	**Theor. mass [M+H]$^+$**	**Experimental mass [M+H]$^+$**	**Mass Error (ppm)**	**Experimental mass [M+H]$^+$**	**Mass Error (ppm)**
**PI**	194.1176	194.1170	-3.09	194.1179	1.54
**DI**	158.0964		-	158.0966	1.26
**DI**	146.0964		-	146.0966	1.37
**DI**	133.0648		-	133.0652	3.00

4'-CARBOXY-MEPHEDRONE					
	**Structure**	**In wastewater**		**In pHLM**	
**Chromatogram**	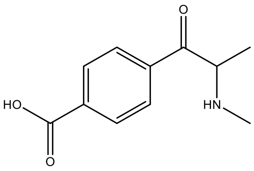	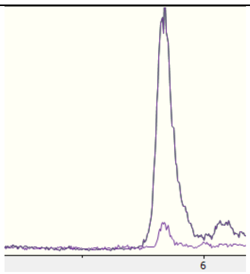		-	
	**Theor. mass [M+H]$^+$**	**Experimental mass [M+H]$^+$**	**Mass Error (ppm)**	**Experimental mass [M+H]$^+$**	**Mass Error (ppm)**
**PI**	208.0968	208.0865	-1.44	-	-
**DI**	144.0808	144.0805	-2.08	-	-

Table S5 Data dependent MS/MS fragmentation (ddMS2) spectra of mephedrone metabolites detected in rat urine sample using LC Q-E.

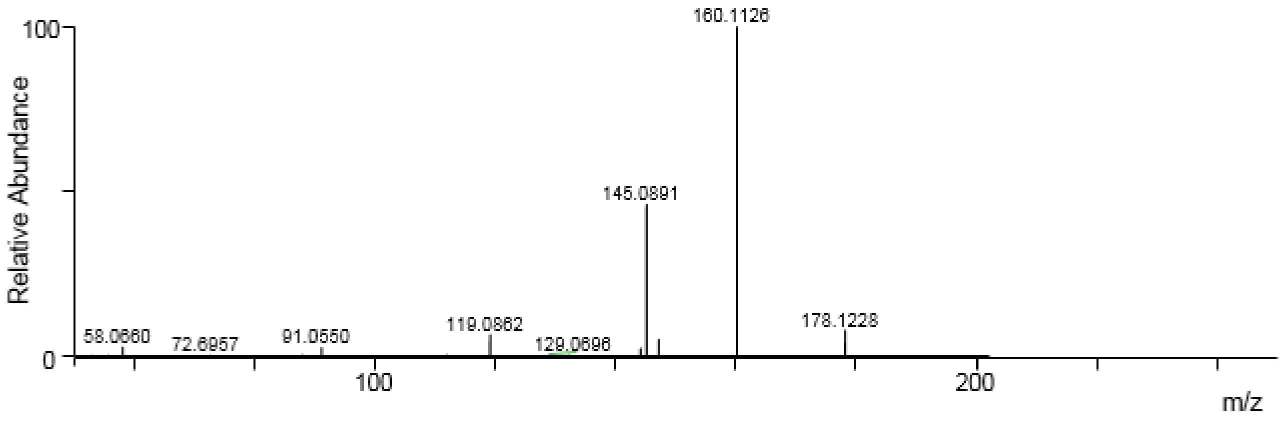	Mephedrone
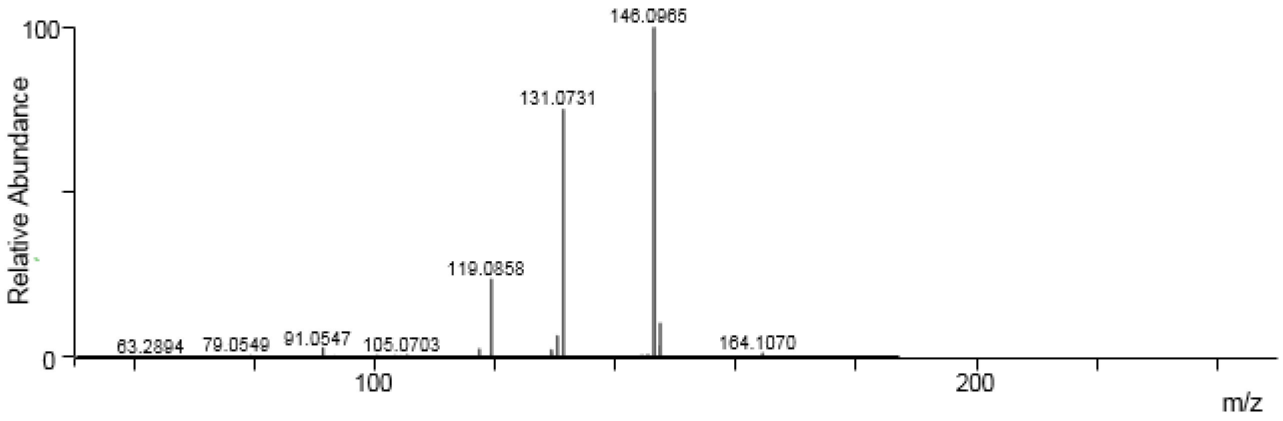	Normephedrone,

<table>
<tr>
<td>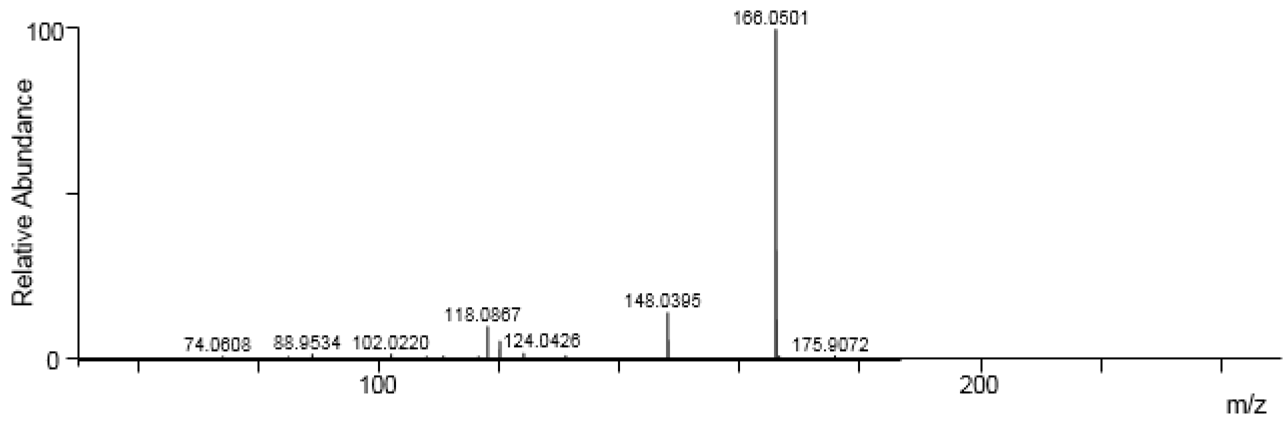
</td>
<td>1-dihydro-
normephedrone</td>
</tr>
<tr>
<td>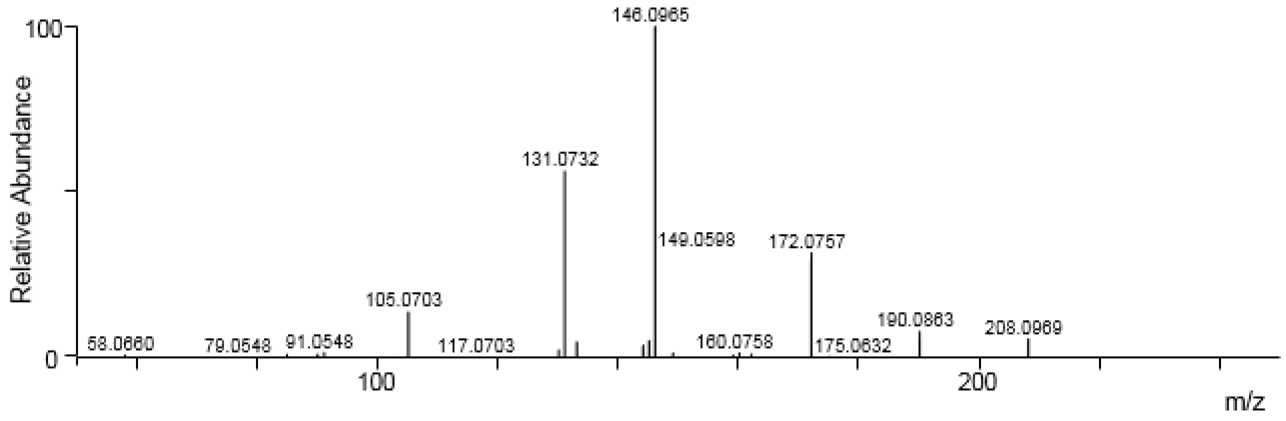
</td>
<td>4’-carboxy-
mephedrone</td>
</tr>
</table>

<table>
<tr>
<td>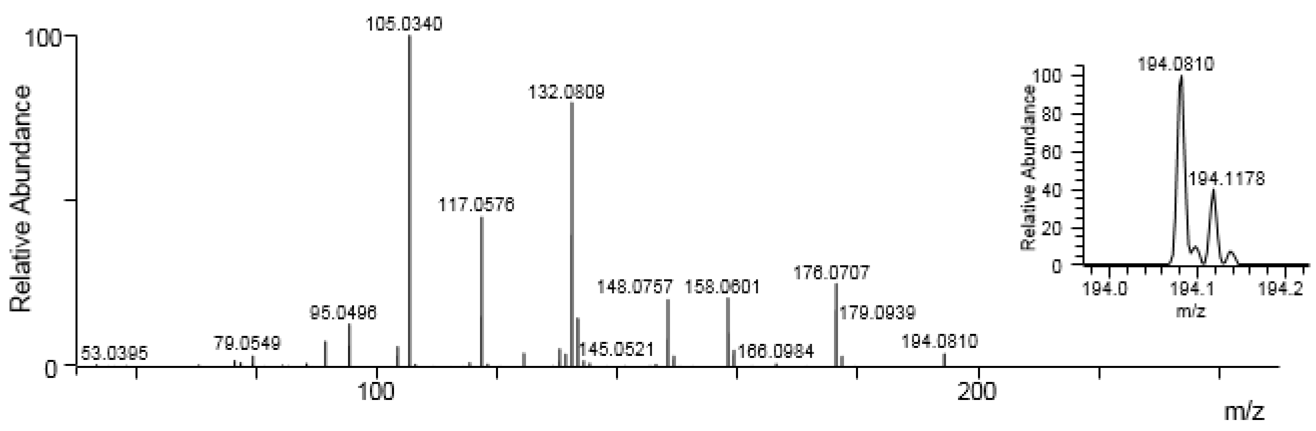
</td>
<td>4’-carboxy-normephedrone, (in the zoom also [M+H]$^+$ of 4’-hydroxy-mephedrone)</td>
</tr>
<tr>
<td>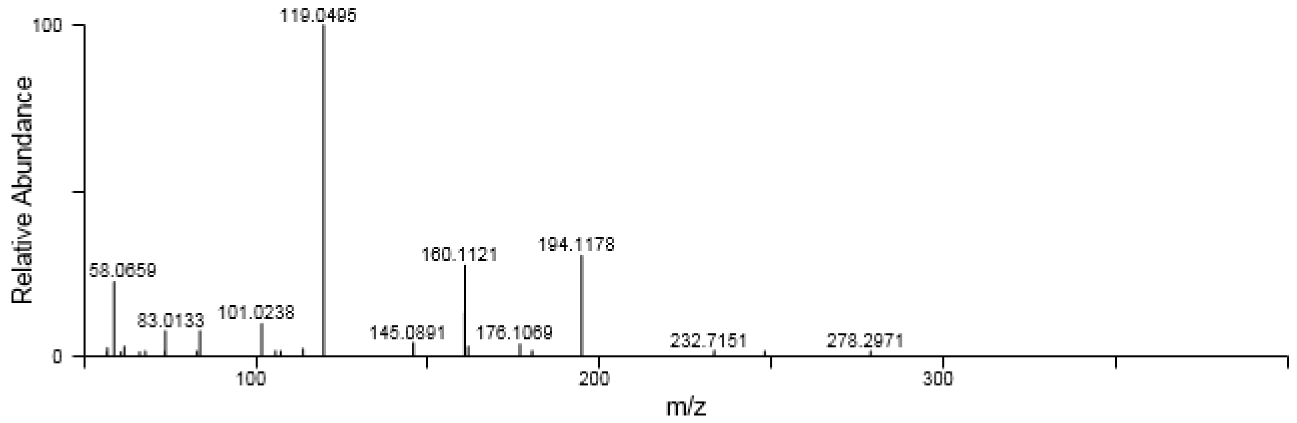
</td>
<td>Hydroxy-mephedrone-3*O*-glucuronide</td>
</tr>
</table>

<table>
<tr>
<td>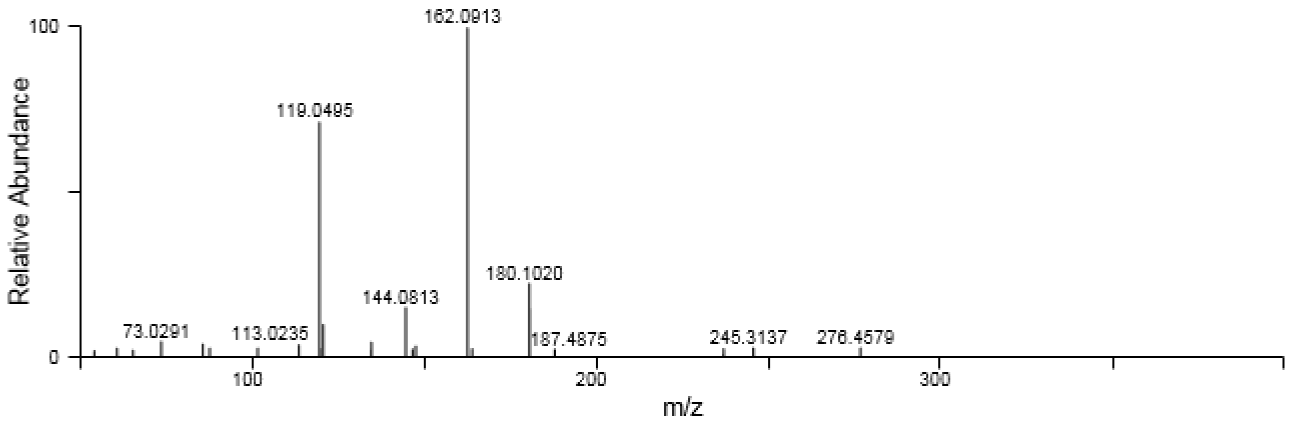
</td>
<td>α-Methyl-HO-normephedrone glucuronide (hydroxy-normephedrone-3*O*-glucuronide)</td>
</tr>
<tr>
<td>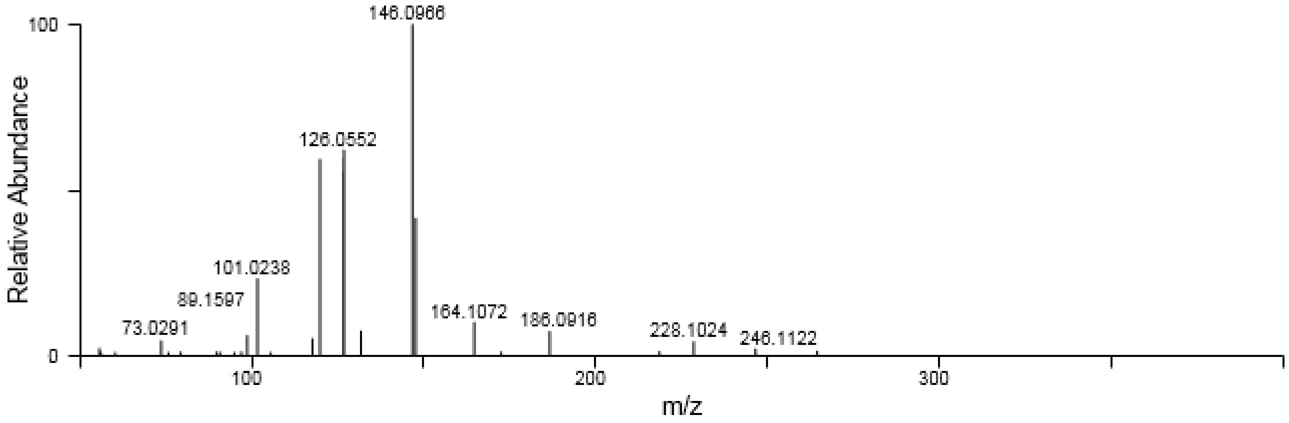
</td>
<td>Normephedrone glucuronide</td>
</tr>
</table>

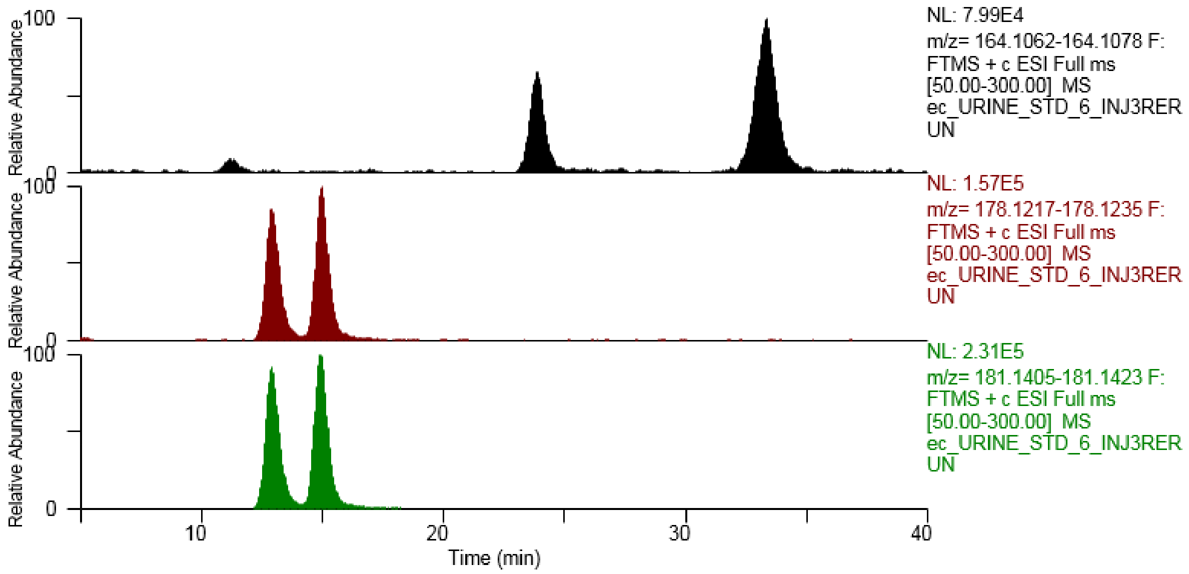

Figure S3 Extracted ion chromatogram of normephedrone (black), mephedrone (red) and mephedrone-D3 (green) in rat urine sample using chiral LC VP

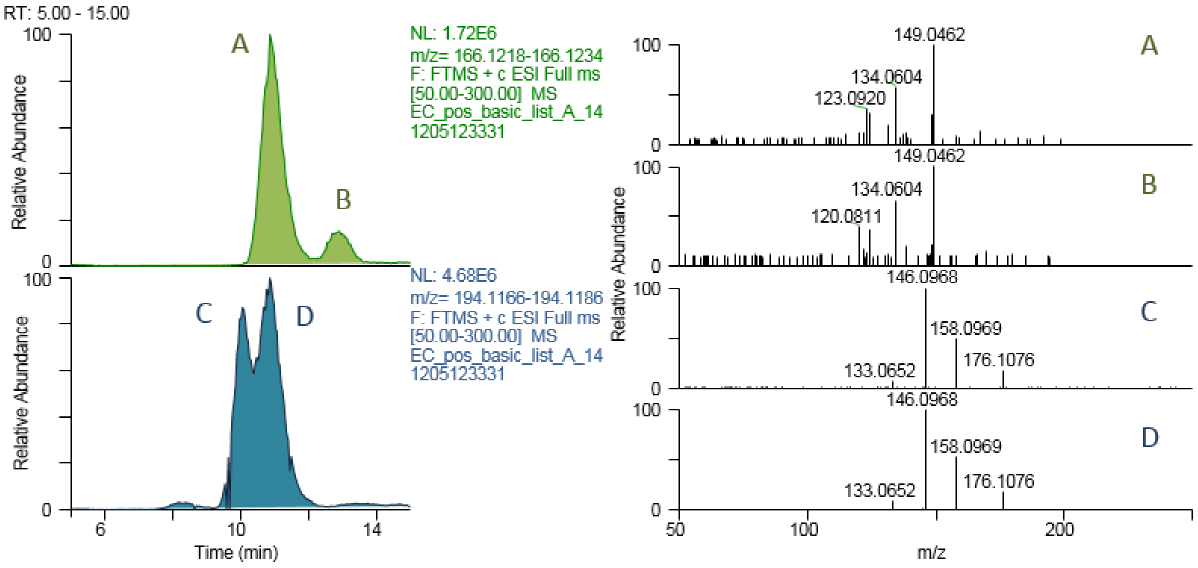

Figure S4 Left: extracted ion chromatograms of 1-dihydro-normephedrone diastereoisomers (top) and partially separated 4'-hydroxy-mephedrone enantiomers (bottom) in rat urine sample using chiral LC VP and corresponding ESI+ (HR) mass spectra (right).

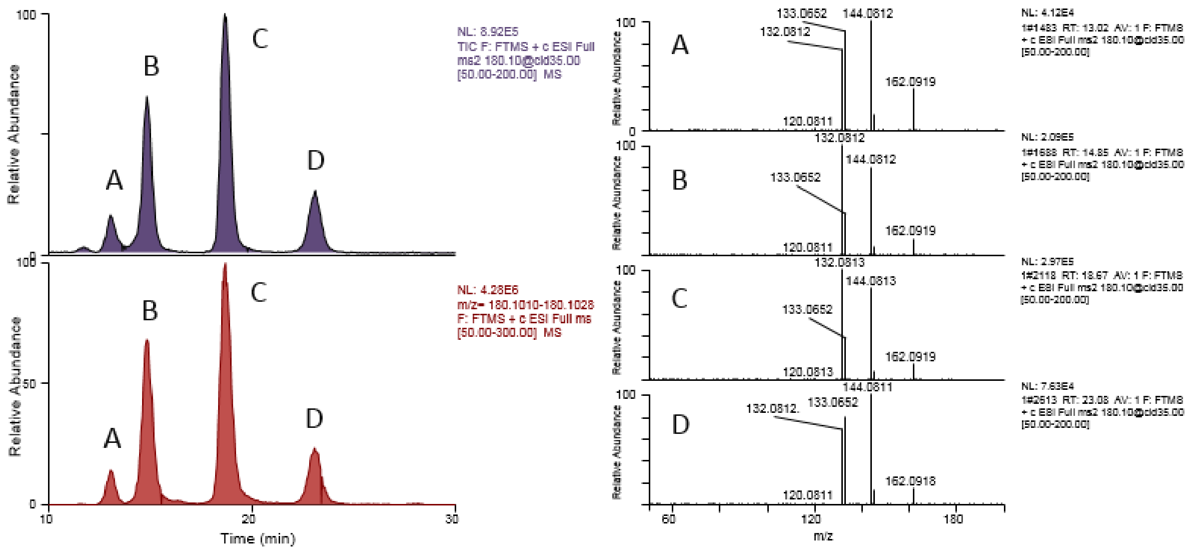

Figure S5 1-Dihydro-4’-oxomethyl-normephedrone enantiomers, labelled as A, B, C and D, were identified in rat urine by chiral LC VP. This compound has two chiral centres, therefore four peaks were detected with identical fragmentation patterns. On the left: top: total ion chromatogram of the nominal mass precursor, bottom: HR extracted ion chromatogram of the full scan. On the right: corresponding ESI+ (HR) mass spectra.

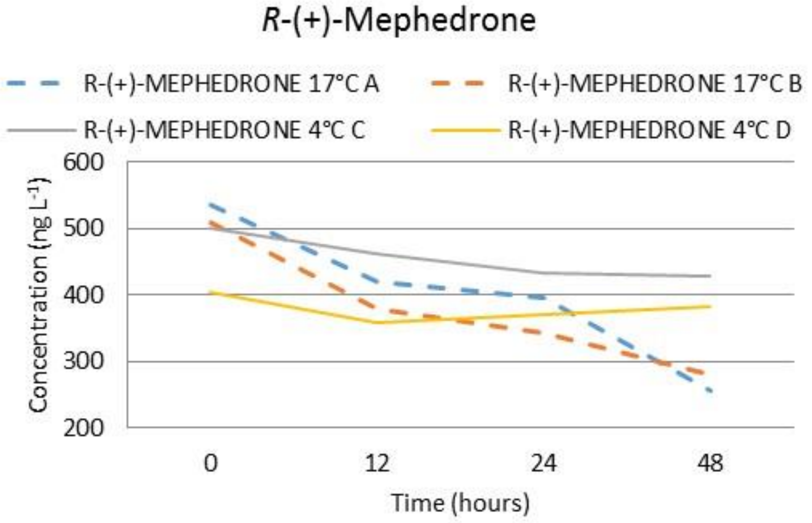
R-(+)-Mephedrone
R-(+)-MEPHEDRONE 17°C A
R-(+)-MEPHEDRONE 17°C B
R-(+)-MEPHEDRONE 4°C C
R-(+)-MEPHEDRONE 4°C D
Concentration (ng L-1)
600
500
400
300
200
0
12
24
48
Time (hours)

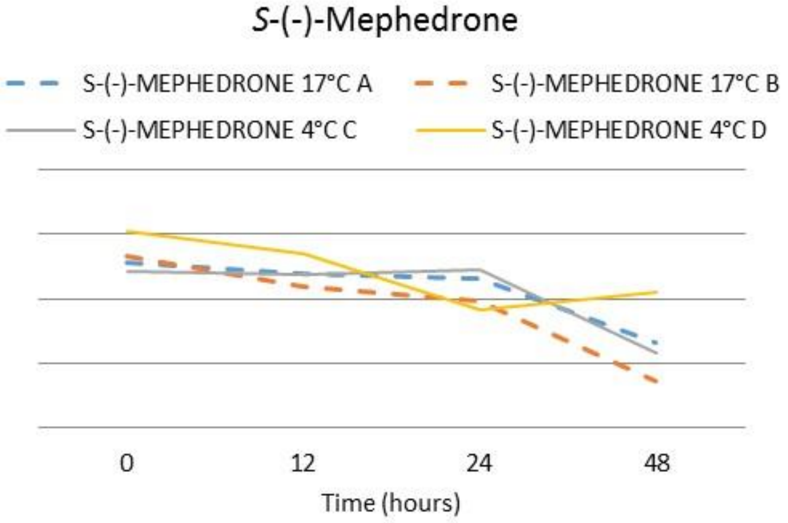
S-(-)-Mephedrone
S-(-)-MEPHEDRONE 17°C A
S-(-)-MEPHEDRONE 17°C B
S-(-)-MEPHEDRONE 4°C C
S-(-)-MEPHEDRONE 4°C D
0
12
24
48
Time (hours)

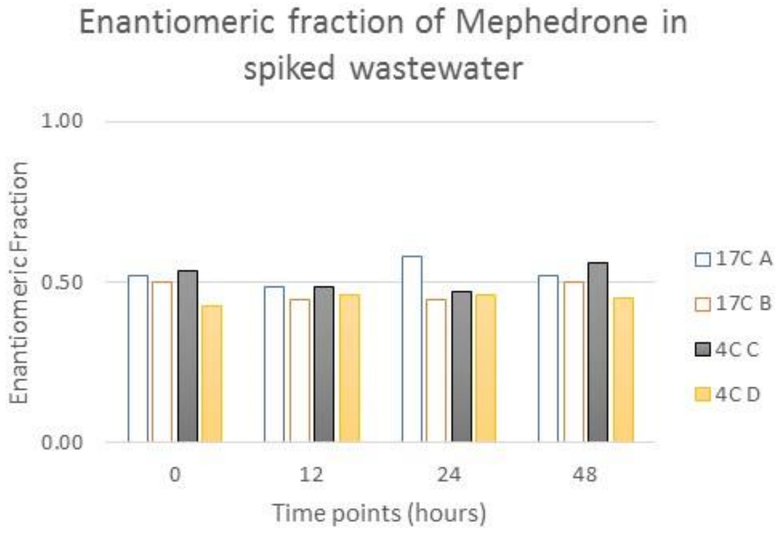
Enantiomeric fraction of Mephedrone in spiked wastewater
Enantiomeric Fraction
1.00
0.50
0.00
0
12
24
48
Time points (hours)
17C A
17C B
4C C
4C D

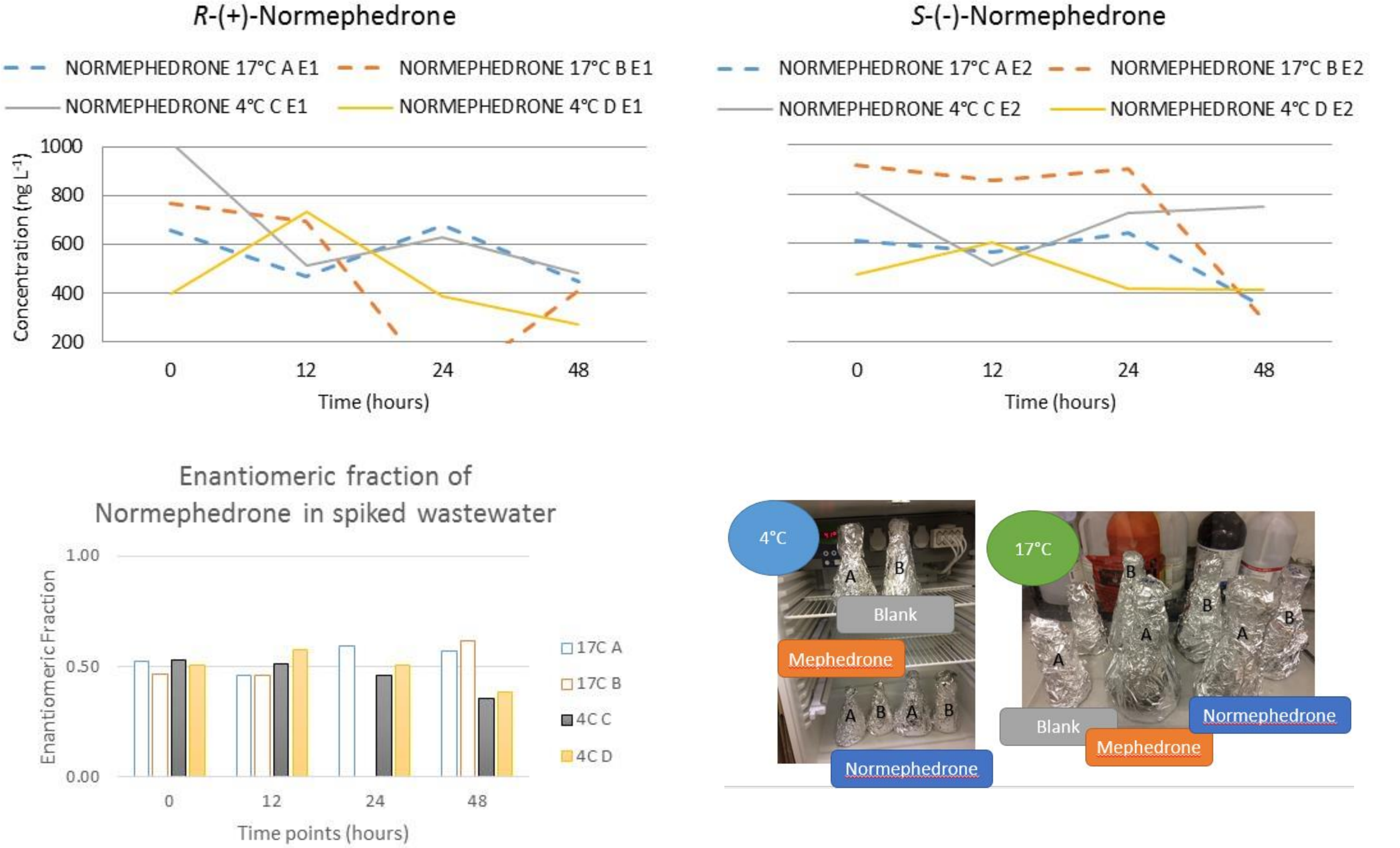

Figure S6 Mephedrone and normephedrone concentrations and enantiomeric fraction in wastewater stability study. Picture of experimental settings.

Table S6 Selected analytes and their properties (MW molecular weight, Exp experimental, Pred predicted, [a] predicted using ACD/labs software (http://www.chemspider.com).

Compound	CAS	Formula	MW	LogP		LogD[a]		Purity (%)	Supplier
				Exp.	Pred.[a]	pH 5.5	pH 7.4		
(±)-Mephedrone	[illegible]189726-[illegible]2-4	$C_{11}H_{15}NO$	177.7	-	1.86±0.31	-0.03	1.55	99.8	Sigma-Aldrich (Cerilliant product)
(±)-Normephedrone	[illegible]941-17-9	$C_{10}H_{13}NO$	163.4	-	-	-	-	98.0	Cayman Chemical Company
(±)-Mephedrone-D_3	[illegible]189972-[illegible]9-9	$C_{11}H_{12}NOD_3$	180.7					99.4	Sigma-Aldrich

S1-Experimental settings, procedure for acetylation of rat urine sample and results.

It was hypothesised that the metabolic hydroxylation reaction may have occurred at different sites of the molecule. For this purpose, acetylated rat urine was injected in LC VP and gas chromatograph coupled to mass spectrometer (GC-MS) systems for suspected acetylated metabolites.

A blank and a positive rat urine sample underwent solid-phase extraction (SPE) with Isolute hydrophilic cation exchange (HCX) cartridges (130 mg, 3 mL, Biotage, Uppsala, Sweden). Cartridges were conditioned with 1mL methanol and 1mL of deionised water. 1 mL of rat urine sample spiked with 10 µL of 1 µg mL^{-1} of mephedrone-D_3 was loaded onto the cartridge. The washing step was carried out with 1mL of deionised water, 1mL 0.01 M HCl followed by 1mL of deionised water. The neutral fraction was obtained after eluting the cartridges with 2 mL of methanol, and the basic fraction with 1mL methanol/NH_3 33% mix 98: 2 v/v. The extracts were evaporated to dryness under nitrogen at 40°C and re-dissolved in 100 µL of methanol. 50 µL were transferred to other vials and dried under nitrogen flow at 40 °C. For the analysis in GC-MS, rat urine was acetylated with 100 µL of a mixture acetic acid/pyridine 3:2 through microwave irradiation for 5 minutes at 450 W. After evaporation, the sample was reconstituted in 50 µL of methanol and then injected in a splitless injection mode into a GC-MS system. The analysis was performed using a Hewlett Packard (HP, Agilent, Waldbronn, Germany) 5890 Series II gas chromatograph combined with an HP 5972A MSD mass spectrometer and an HP MS ChemStation (DOS series) with HP G1034C software version C03.00. The column was Thermo Scientific TG-1MS capillary (12 m × 0.2 mm I.D.), cross-linked methyl silicone, 330 nm film thickness. The following GC conditions were set: injection port temperature at 280 °C, helium as carrier gas, 1mL min^{-1} as flow rate. The column temperature was programmed from 100 to 310 °C at 30 °C min^{-1}, initial time 2 min, final time 5 min. The MS conditions were as follows: electron ionization (EI) mode, 70 eV as ionization energy, ion source temperature at 220 °C and capillary direct interface heated at 280 °C. The acquisition was in full scan mode with m/z range from 50 to 550 uma.

However, due to the presence of co-eluting interferences, no distinguishable peaks for the enantiomers were found. No spectra corresponding to the (1-2 acetyl) hydroxyl-normephedrone were recorded.

Table S7 Experimental set up of the reactors used for (a) incubating wastewater and (b) pHLM.

(a)

	Wastewater reactors			
	Biotic	Abiotic	Clean	Control
Mephedrone (100 ng mL^{-1})	x	x	x	
NaN_3		x	x	
Demineralised water			x	
Wastewater	x	x		x

(b)

	Biological pHLM reactors		
	Incubation	NoHLM	NoRegSys
Phosphate buffer pH 7.4	x	x	x
Regenerating system (Reg Sys)	x	x	
Substrate solution	x	x	x
SOD	x	x	x
HLM	x		x

S2-Setup for pHLM incubation.

Experiment A. (±)-Mephedrone was incubated at a concentration of 2.5 µM over 60 minutes in triplicate biological reactors. The reactors are described in Table S7b. 100 µL of each reactor contained 90 mM of phosphate buffer at pH 7.4, 25 µM of substrate solution and 20 U mL-1 of Superoxide Dismutase (SOD). Apart from the "No HLM" reactor, 1 mg mL-1 of pooled human liver microsomes (pHLM) (Corning, The Netherlands) was added to all reactors. The regenerating system consisted of isocitrate, $MgCl_2$ and NADP+ (Biomol, Germany). Sampling points were set at 0, 10, 20, 30 and 60 minutes. The reaction was commenced with the addition of pHLM (37 °C) and it was stopped at each specified time points with the addition of ice cold acetonitrile containing IS at a concentration of 100 ng mL-1. Samples were shaken thoroughly and left in the freezer for 5 minutes. LLE was performed with 300 µL ethyl acetate (pH 8-9 adding sodium phosphate). Samples were then centrifuged for 5 minutes at 14680 rpm. The supernatant was gently evaporated to dryness under nitrogen flow. Finally, samples were reconstituted in 55 µL of 1mM ammonium acetate/methanol 85:15 and 5 µL were injected in chiral LC VP system.

Experiment B. Mephedrone was incubated at a final concentration of 10 µM over 180 minutes in biological duplicates. A single reactor consisted of: analyte solution, a buffer solution, containing 50 mM KH_2PO_4 and 5 mM $MgCl_2$ at pH 7.4, a NADPH 50 mM solution and pHLM. Glucuronic acid and PAPS were used as substrates for the investigation of Phase II metabolism reactions, such as glucuronidation and sulfation respectively. Sampling points were set at 0, 10, 20, 30, 60 and 180 minutes for mephedrone incubation and at 180 minutes for phase II metabolism investigation. In this experiment, the blank contained the analyte but not the pHLM. IS was also added in the ice cold acetonitrile to all the incubated samples at the end of the reaction. Samples were shaken thoroughly and centrifuged for 10 minutes at 12000 rpm. Samples for the investigation of the phase II metabolism were evaporated at 40 °C and reconstituted in 100 µL of water/methanol 8:2. Ten microliters were injected in the liquid chromatograph coupled with quadrupole-time of flight (LC QTOF) system. LLE of the pHLM samples incubating normephedrone was performed with 300 µL ethyl acetate (pH 8-9 adding sodium phosphate). Samples were then centrifuged for 5 minutes at 14680 rpm. The supernatant was evaporated to dryness under nitrogen flow. Samples were reconstituted in 500 µL of 1mM ammonium acetate/methanol 85:15 v/v. 20 µL were injected in the chiral LC TQD system.

S3-LC and MS source setting in the systems used.

TQD.

MS setting. The system operated with a capillary voltage of 3 kV, source temperature at 150 °C, desolvation temperature at 265 °C and desolvation gas flow at 550 L h^{-1}. Nitrogen, supplied by a high purity nitrogen generator (Peak Scientific, UK), was used as a nebulising and desolvation gas. Argon (99.999%) was used as a collision gas.

LC-Q-E.

LC condition. Mobile phase A was a solution of 2 mM aqueous ammonium formate plus 0.1% formic acid at pH 3, whilst mobile phase B was a solution of 2 mM aqueous ammonium formate with acetonitrile:methanol (50:50, v/v; 1% water) plus 0.1% formic acid. The sample injection volume was 10 μL. The flow rate was set at 0.5 mL min^{-1} for 10 min and at 0.8 mL min^{-1} from 10 to 13.5 min. The mobile phase gradient was as follows: 0–1.0 min 99% A, 1–10 min to 1% A, 10–11.5 min hold 1% A, 11.5–13.5 min hold 99% A.

MS setting. Source spray voltage was at 3 kV (positive polarity) and at −4 kV (negative polarity); heater temperature and ion transfer capillary temperature were both set at 320 °C; S-lens RF level was at 60.0; sheath and auxiliary gases were 60 and 10 arbitrary units, respectively.

LC-QTOF.

LC condition. The injection volume of the sample was 10 µL. The chosen mobile phases delivered at 0.4 mL min-1 were: A – 1mM NH4F in MilliQ-water; B – MeOH. The gradient was set as follows: 0-3 min 5% B, 3-4 min 5-60% B, 4-14 min 60% B, 14-14.1 min 60-98% B, 14.1-17 min 98% B, 17-17.1 min 98-5% B, 17.1-20 min 5% B.

MS setting. The capillary was set at 4500 V, the nebulizer gas at 3.0 bar, the dry gas at 11.0 L min^{-1} and the dry temperature at 220°C.

Table S8 MRM transitions in chiral LC-TQD method

**Compound**	**CV/ CE[a]**	**MRM1 (quantification)**	**CV/ CE[a]**	**MRM2 (confirmation)**	**CV/ CE[a]**	**MRM3 (confirmation)**	**MRM1/ MRM2 ratio ± SD**	**MRM1/ MRM3 ratio ± SD**	**Internal standard**
Mephedrone	10/12	178.1 > 160.1	10/22	178.1 > 145.0	10/22	178.1 > 119.0	1.6 ± 0.2	8.5 ±2.1	Mephedrone-D_3
Normephedrone	10/20	164.0 > 131.0	10/32	164.0 > 91.0	-	-	6.6 ± 0.8	-	Mephedrone-D_3
Mephedrone-D_3	30/22	181.1 > 163.1	-	-	-	-	-	-	-

[a]CV, cone voltage (V); CE, collision energy (eV)

Table S9 Method validation parameters (chiral LC-TQD) for mephedrone and normephedrone.

(a)

Compound	R_t[a] (min)	Rel. R_t[a]	Sample diluent: Linear range (µg L^{-1})	R^2	$IDL_{S/N}$[b] (µg L^{-1})	$IQL_{S/N}$[c] (µg L^{-1})	WWTP influent: MDL[d] (ng L^{-1})	MQL[e] (ng L^{-1})
R-(+)-Mephedrone	16.5 ±0.4	0.3	0.25-500	0.9990	0.25	0.50	1.30	2.60
S-(-)-Mephedrone	21.0 ±0.5	0.2	0.25-500	0.9993	0.25	0.50	0.66	2.63
R-(+)-Normephedrone	44.2 ±0.8	6.5	0.25-500	0.9915	0.25	5.0	1.35	26.9
S-(-)-Normephedrone	68.8 ±1.4	6.7	0.25-500	0.9911	0.25	5.0	1.35	27.0

(b)

SPE recovery % (n=3)	25 ng/L	250 ng/L	2500 ng/L
R-(+)-Mephedrone	109.1 ± 3.2	99.3 ± 4.8	80.7 ± 7.0
S-(-)-Mephedrone	99.0 ± 8.5	99.1 ± 4.3	87.2 ± 11.5
R-(+)-Normephedrone	72.8 ± 1.3	97.4 ± 9.2	108.5 ± 5.7
S-(-)-Normephedrone	79.4 ± 0.8	86.0 ± 2.0	113.2 ± 1.4

(c) Method precision; D represents day

Intra-day RSD% (n=4)	5 ng L^{-1} D 1	5 ng L^{-1} D 2	5 ng L^{-1} D 3	50 ng L^{-1} D 1	50 ng L^{-1} D 2	50 ng L^{-1} D 3	500 ng L^{-1} D 1	500 ng L^{-1} D 2	500 ng L^{-1} D 3
R-(+)-Mephedrone	9.8	13.7	14.1	3.6	6.8	14.6	3.7	10.0	5.6
S-(-)-Mephedrone	10.7	12.0	4.6	5.2	12.9	8.4	9.2	3.7	2.8
R-(+)-Normephedrone	14.1	20.8	13.3	2.8	13.3	8.3	1.5	5.5	12.8
S-(-)-Normephedrone	18.2	11.2	3.2	17.4	18.9	0.5	13.9	7.9	18.5

Inter-day RSD% (n=3)	5 ng L^{-1}	50 ng L^{-1}	500 ng L^{-1}
R-(+)-Mephedrone	12.5	8.3	6.4
S-(-)-Mephedrone	9.1	8.8	5.2
R-(+)-Normephedrone	10.3	8.1	6.6
S-(-)-Normephedrone	11.6	12.3	13.4

(d) Instrumental precision; D represents day

Intra-day RSD% (n=4)	5 µg L^{-1} D 1	5 µg L^{-1} D 2	5 µg L^{-1} D 3	50 µg L^{-1} D 1	50 µg L^{-1} D 2	50 µg L^{-1} D 3	500 µg L^{-1} D 1	500 µg L^{-1} D 2	500 µg L^{-1} D 3
R-(+)-Mephedrone	9.3	6.7	5.5	1.9	5.7	5.4	2.9	5.5	4.4
S-(-)-Mephedrone	3.5	6.7	1.1	3.6	2.5	2.7	9.3	4.3	2.2
R-(+)-Normephedrone	24.5	5.4	9.2	11.5	8.0	8.2	3.7	3.0	2.7
S-(-)-Normephedrone	27.5	12.4	1.1	5.6	4.5	6.6	4.3	2.1	4.0

Inter-day RSD% (n=3)	5 µg L^{-1}	50 µg L^{-1}	500 µg L^{-1}
R-(+)-Mephedrone	7.1	4.3	4.3

S-(-)-Mephedrone	3.8	3.0	5.2
R-(+)-Normephedrone	8.5	9.2	3.1
S-(-)-Normephedrone	7.6	5.6	3.5

(e)

	R_s[f]	EF[g]		
		5 μg L^{-1}	50 μg L^{-1}	500 μg L^{-1}
Mephedrone	1.4 ±0.1	0.50±0.0	0.50±0.0	0.48±0.0
Normephedrone	4.0 ±0.4	0.51±0.0	0.50±0.0	0.50±0.0

[a] Retention time

[b] Instrumental Limit of Detection (IDL). It was determined at a concentration value giving a signal-to-noise ratio (S/N) ≥ 3 for all the MRM transitions selected for cocaine.

[c] Instrumental Limit of Quantification (IQL). It was determined at the minimum concentration value giving S/N ≥ 10 for all the MRM transitions.

[d] Method Detection Limit (MDL).

[e] Method Quantification Limit (MDL).

[f] Enantiomeric resolution.

[g] Enantiomeric fraction.

S4-Absolute configuration determination of mephedrone using circular dichroism (CD) and computational study

Due to lack of analytical standards of single mephedrone enantiomers, elution order of the peaks needed to be confirmed under used chromatographic conditions. The assignment of the peaks was only possible through CD analysis. Absolute configuration determination of mephedrone was undertaken using a Perkin Elmer Series 200 HPLC system (equipped with a temperature controlled autosampler and column compartment, pump and a UV/VIS detector) coupled with a Chirascan Circular Dichroism Spectrometer (Applied Photophysics) equipped with a quartz spectrophotometer cell type 585.3/Q/10 cuvettes with a path length of 10 mm for micro flow (Starna Scientific). The operating conditions are given in Table S10. Separation of mephedrone enantiomers was achieved as described Castrignanò et al. The background, represented by the mobile phase, was subtracted from CD spectra. UV absorbance and CD spectra were acquired simultaneously at αmax= 265 nm (Maskell, De Paoli et al. 2011) (Figure S7). The predicted UV data were slightly shifted in the spectrum obtained from computational study due to solvation effects on the electronic transitions when compared with the UV mephedrone spectrum reported by Maskell et al. (2011) (Maskell, De Paoli et al. 2011). Computational study was performed with ArgusLab 4.0 (Mark A. Thompson, Planaria Software LLC, WA, USA) and pre-optimised structures with AM1 using x-Ray coordinated as starting geometry. ZINDO-RPA was used to predict the UV-vis and CD transitions for (±)-mephedrone (Figure S8a) and for (±)-normephedrone (Figure S8b). In correspondence of the first maximum absorbance peak at 265 nm the first eluting mephedrone enantiomer rotated the plane of polarized light with a negative Cotton effect, whilst the second peak with a positive effect. By combining the information obtained from the experimental spectrum and the modelling study, it was possible to confirm that *R*-(+)-mephedrone eluted as the first enantiomer, while *S*-(-)-mephedrone as the second. Due to similar behaviour of the metabolite, also *R*-(+)-normephedrone and *S*-(-)-normephedrone were assessed as the first and second eluting enantiomers under the chromatographic conditions used.

Table S10 Operating conditions for the absorbance and CD spectra of (±)-mephedrone

Wavelength	265 nm
Spectral bandwidth	1 nm
Step size	1 nm
Concentration	170 µg mL^{-1}
Solvent	1mM ammonium acetate/methanol 85:15
Time	1000 s
Points	10000
Samples	4000
Temperature (QNWPeltier)	25.02 ºC

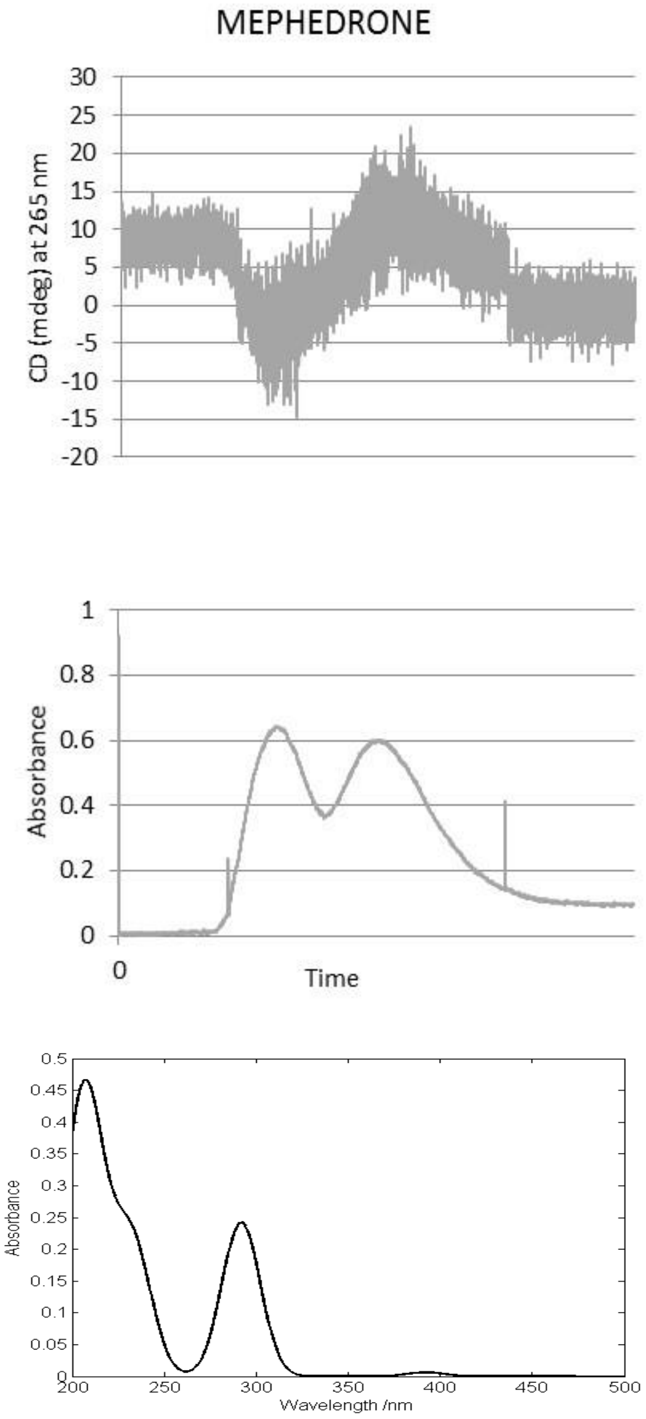

(a)

(b)

(c)

Figure S7 CD and absorbance spectra of (±)-mephedrone (a,b). UV spectra of (±)-mephedrone from the computational study (c).

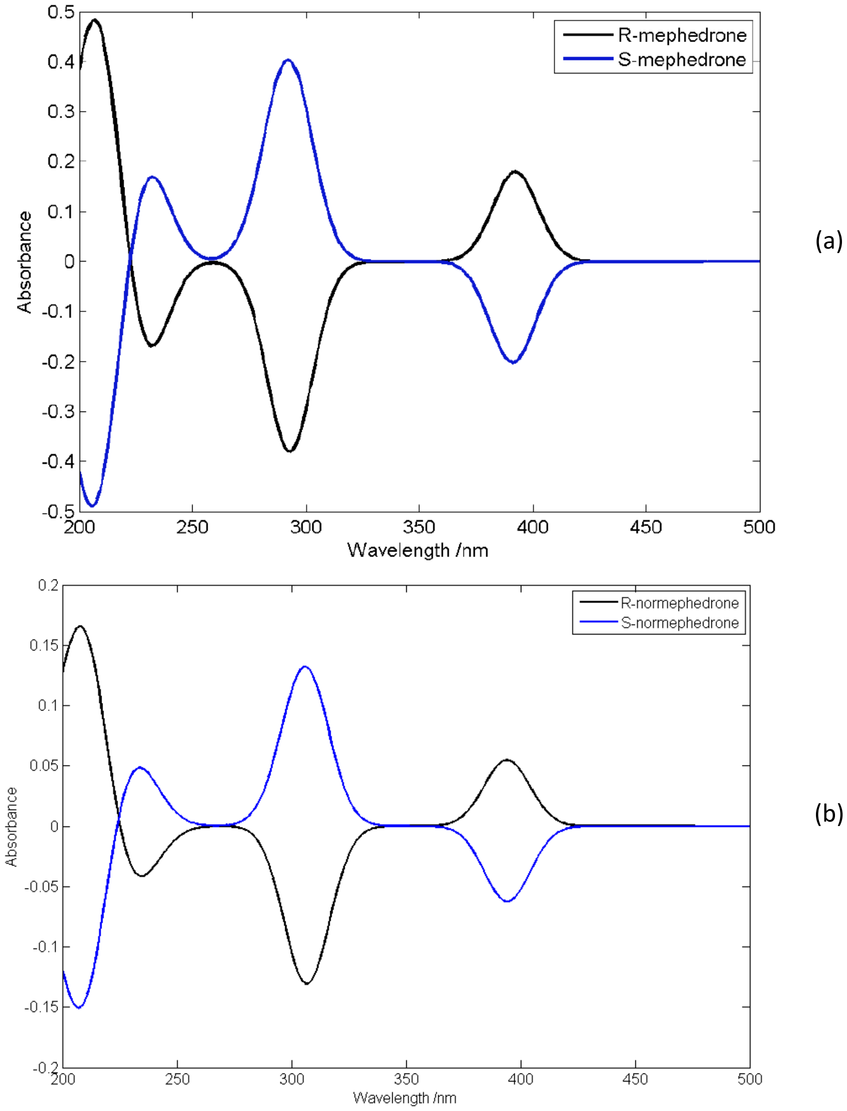

Figure S8 Predicted CD spectra for (±)-mephedrone (a) and for (±)-normephedrone (b).
